# The interaction between common genetic mutations in AML and the immune landscape: mechanisms and implications for immune response

**DOI:** 10.3389/fimmu.2025.1635111

**Published:** 2025-08-11

**Authors:** Xuege Guo, Hanlu Zhang, Xiao Wang, Lijuan Li, Liansheng Zhang

**Affiliations:** ^1^ Department of Hematology, The Second Hospital and Clinical Medical School, Lanzhou University, Lanzhou, China; ^2^ Gansu Provincial Hematology Clinical Medical Research Center (National Branch), The Second Hospital of Lanzhou University, Lanzhou, China

**Keywords:** acute myeloid leukemia, gene mutation, immune microenvironment, risk stratification, immunotherapy, neoepitopes

## Abstract

Acute myeloid leukemia (AML) is a heterogeneous hematologic malignancy driven by diverse genetic mutations that shape tumor progression, immune evasion, and clinical outcomes. While molecular profiling has improved AML classification, the precise impact of specific mutations on immune cell infiltration and dysregulation remains insufficiently understood. This review examines the immunologic consequences of common AML mutations—including *FLT3-ITD*, *NPM1*, *DNMT3A*, *TP53*, *IDH1/2*, and *NRAS*—and their role in remodeling the immune microenvironment. We further explore the dynamic shifts in immune responses across different AML risk stratifications, emphasizing the balance between immune activation and suppression, which is influenced by specific genetic alterations. Additionally, we highlight the emerging potential of immunotherapies targeting neoepitopes derived from driver mutations, offering promising avenues to overcome immune escape and enhance anti-tumor immune responses. By integrating genetic mutations and immunologic insights, this review outlines a framework for developing more precise and effective immunotherapies for AML.

## 1 Introduction

Acute myeloid leukemia (AML) is a highly aggressive hematologic malignancy that accounts for approximately 80% of all acute leukemias in adults (1). The pathogenesis of AML is complex, involving a combination of genetic mutations, chromosomal abnormalities, and environmental factors, all of which lead to abnormal hematopoietic stem cell development. These aberrant cells proliferate uncontrollably, progressively replacing normal hematopoietic tissue and impairing the production of healthy blood cells. This disruption manifests clinically as anemia, bleeding, and increased susceptibility to infections (2). In 1976, the French-American-British (FAB) Cooperative Group, consisting of hematology experts from France, the United States, and the United Kingdom, first proposed diagnostic and classification criteria for AML (FAB classification), dividing AML into eight subtypes (M0 to M7). Accurate classification of leukemia is essential for selecting appropriate treatment strategies. However, the FAB classification, which is based primarily on the morphology, differentiation status, and chemical staining of bone marrow (BM) leukemia cells, has become increasingly insufficient to meet the demands of modern clinical diagnosis and treatments. In response, the World Health Organization incorporated molecular genetic, molecular biology, and immunological characteristics into the classification of AML for the first time in its 2001 “Classification of Hematopoietic and Lymphoid Tumors”. This revision aimed to provide a more biologically relevant classification system and was updated in 2008 and 2016 based on new research findings. Unlike the morphology-based FAB classification, the World Health Organization classification places greater emphasis on the role of genetic mutations and chromosomal abnormalities in AML classification, particularly in the context of prognosis and risk stratification (3). Today, the cytogenetic and molecular characteristics of AML are not only fundamental to accurate disease classification but also crucial for guiding clinical decision-making. Through the detection of specific genetic mutations and chromosomal abnormalities, clinicians can more precisely predict outcomes such as the complete remission (CR) rate, disease-free survival, relapse risk, and overall survival (OS), enabling the development of personalized treatment regimens tailored to individual patients (2).

In the initiation and progression of AML, the tumor microenvironment (TME) is no longer a passive “bystander”. It functions both as an “inhibitor”, slowing leukemogenesis by impeding the proliferation of malignant cells, and as a potent “catalyst”, playing a critical role in sustaining and promoting leukemia development. The TME is a complex network composed of immune cells, cytokines, extracellular matrix components, and other immune-regulatory molecules, with its dynamic alterations directly influencing tumor progression and therapeutic responses (4). Recent studies have highlighted the crucial role of genetic mutations in AML in shaping and regulating the immune microenvironment. These mutations impact immune cell infiltration patterns and immune function via various pathways, exhibiting significant heterogeneity. For instance, mutations associated with favorable prognosis, such as *NPM1* mutations, are typically linked to immune activation and enhanced anti-tumor responses (5), while mutations associated with poor prognosis, such as *TP53* mutations, often lead to immune suppression or escape, impairing immune surveillance (6). Importantly, the AML immune microenvironment shows both immune activation and suppression at the same time. Immune cells may become dysfunctional in suppressive conditions, but can also be reactivated by signals from the tumor. This balance between immune response and escape reflects the complexity of AML and varies with different genetic mutations.

Although significant progress has been made in genetic research on AML, which has gradually been incorporated into risk stratification systems, a comprehensive understanding of how gene mutations influence immune cell infiltration, the expression of immune regulatory molecules, and immune evasion mechanisms remains lacking. This review aims to explore how common genetic mutations in AML shape the immune microenvironment through various mechanisms and how these alterations impact patient prognosis.

## 2 AML tumor cell remodeling of the immune microenvironment

The immune system in AML patients exhibits significant heterogeneity and dysfunction, with both the innate and adaptive immune systems being suppressed and dysregulated. In the context of the innate immune system, AML patients show a marked reduction in the number of natural killer (NK) cells, particularly in the CD56dimCD16+ functional subset (7). The imbalance between immature and overmature NK cell subpopulations varies significantly among individuals (8, 9), which may be linked to specific genetic mutations. Furthermore, tumor cells further impair NK cell function by downregulating the activating ligand HLA-E, secreting soluble ligands such as MICA/B (10), and upregulating inhibitory receptors like TIM-3, KIR, and CD159a (8–11). As the number of regulatory T cells (T_regs_) increases, NK cell dysfunction becomes more pronounced. Despite the widespread expression of ligands for the NK cell-activating receptor NKG2D on AML cells (12), NK cell responses to cytokine stimulation remain diminished, as evidenced by significantly reduced expression of granzyme B and IFN-γ (13). Additionally, macrophages in AML patients undergo a phenotypic shift from the anti-tumor M1 type to the immunosuppressive M2 type. M2 macrophages further promote the immunosuppressive environment through the high expression of inhibitory receptors (14). Regarding dendritic cells (DCs), although the overall number of DCs is increased, the conventional dendritic cell type 1 (cDC1) subset is significantly reduced (15, 16), indicating dysfunction within this population.

There are significant individual differences in lymphocyte counts among AML patients. In some cases, the lymphocyte count is approximately five times higher than normal, while in others, it remains within the normal range (1). Furthermore, lymphocyte distribution shows heterogeneity, with a slightly lower proportion in the BM and a slight increase in the peripheral blood (PB), though there are no significant changes in relative proportions (15, 17). T cell function is significantly impaired in AML patients. In general, T cells exhibit reduced proliferative capacity (18–21), increased apoptosis (19, 22), diminished expression of costimulatory molecules (19), and upregulated expression of inhibitory receptors (15, 17, 19, 23–25) often with increased co-expression of these receptors (19, 26, 27). These changes lead to a decrease in the secretion of pro-inflammatory cytokines, such as IFN-γ, TNF-α, and IL-2 (21), thus weakening the anti-tumor immune response. Although studies have shown that the number of CD8+ T cells in the BM is elevated (19, 24), and that these cells are predominantly effector memory T cells (T_em_) (17), their functionality remains compromised. In the PB, there is an increase in the proportion of terminally differentiated effector cells, while the proportion of naive T cells (T_n_) decreases (19, 21, 28). However, in a study by Oscar Brück, it was found that compared to healthy individuals, T cells in the BM of AML patients exhibit high expression of PD-1 and low expression of LAG-3 and TIM-3 (20). This suggests that the immunological characteristics of T cells in AML may be influenced by multiple factors. Additionally, T cells in the BM of AML patients show impaired immune synapse formation, with reduced F-actin polymerization and insufficient recruitment of signaling molecules (19). This may be related to the dysfunction of AML cells as antigen-presenting cells (18). Moreover, AML patients have reduced T helper 1 (Th1) cells and decreased IFN-γ secretion (21), while T helper 17 (Th17) cells are increased and secrete IL-17, promoting AML cell proliferation and inhibiting Th1 differentiation (16, 21). There is also an increase in CD4+ T cells in the BM expressing PD-1+/OX40+, ICOS+ (26), and TIM-3+ (29). Although these CD4+ T cells are partially activated, their function remains relatively weak. In certain patients, there is a notably higher frequency of double-positive T cell subsets in the BM (26). Additionally, the increased number of T_regs_ suppresses the anti-leukemic function of effector T cells (T_effs_) (1, 24), and removing T_regs_ can partially restore T cell functionality (21). Unlike typical NKT cells, CD3+CD56+ T cells in AML patients exhibit significantly reduced cytotoxic potential (18). The BM of AML patients contains atypical B cells (30), although their exact role remains unclear. Overall, the immune microenvironment in AML patients is characterized by immune suppression, which hinders anti-tumor immune responses and promotes tumor immune escape and disease progression.

## 3 Common gene mutations in AML patients and the immune microenvironment

In different patient populations, the immunogenicity of AML cells and the quality of the immune response are shaped by specific oncogenic driver mutations. Even in the presence of the same mutations, variations in co-mutations or other genetic background differences can lead to distinct pathways of AML progression, resulting in differing prognoses. Although AML is typically characterized by a low mutation burden, certain high-frequency driver mutations, such as *FLT3-ITD* and *NPM1* mutations, can generate immunogenic peptides that act as tumor-specific antigens, triggering targeted immune responses (31, 32). Therefore, understanding the impact of these genetic mutations on the immune microenvironment is essential for the development of effective immunotherapy strategies for AML.

### 3.1 *FLT3-ITD* mutation

*FLT3* is a receptor tyrosine kinase predominantly expressed in DCs (33). The *FLT3* signaling pathway regulates the differentiation and mobilization of precursor DCs, as well as the homeostatic division of cDCs in peripheral lymph nodes (29). In patients with AML, approximately 20-25% harbor *FLT3-ITD* mutations, while 5-7% have mutations in the *FLT3-TKD* (34). These mutations lead to constitutive activation of the *FLT3* receptor, resulting in enhanced cell proliferation and inhibition of apoptosis (10).

#### 3.1.1 T cell dysfunction and immune escape in *FLT3-ITD* mutant AML

T cells can specifically recognize *FLT3-ITD*-mutated AML cells and induce cell lysis by secreting IFN-γ, granzyme B, and perforin (16). In patients with *FLT3-ITD* mutations, the proportion of CD3+ T cells (1), including both CD4+ and CD8+ subsets, is significantly increased (35), which contrasts with the significantly reduced percentages of B cells, plasmablasts (1, 36), and NKT cells (37). However, despite the increase in T cells, the anti-leukemia immune response is impaired due to multiple immune escape mechanisms, such as the predominant expansion of T_regs_ among CD4+ T cells. Studies have shown that T_regs_ are significantly enriched in the BM and spleen of *FLT3-ITD*-mutant AML mice (16), suggesting that the increase in CD4+ T lymphocytes is primarily driven by the expansion of T_regs_.

**Figure 1:**
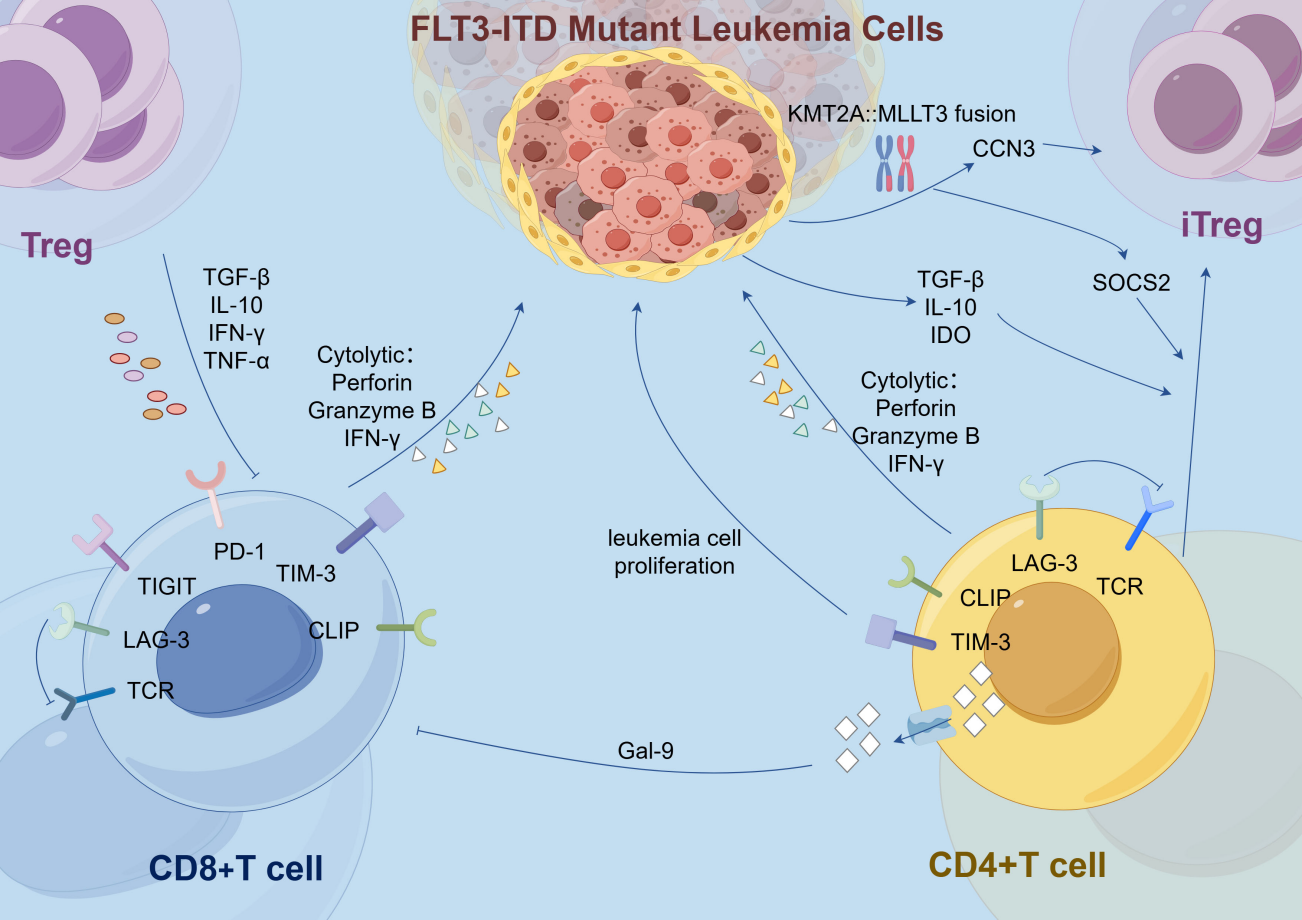
In the *FLT3-ITD*-mutated AML microenvironment, elevated T cell subsets, including CD8+ and CD4+ T cells, are suppressed by overexpression of immune checkpoints (e.g., PD-1, TIGIT, LAG-3, TIM-3) and their ligands (e.g., Gal-9). Additionally, in cells harboring both *FLT3-ITD* mutations and the *KMT2A*::*MLLT3* fusion, activated *CCN3* enhances Treg functionality, while *SOCS2* promotes the polarization of iTregs from CD4+ T cells. *FLT3-ITD*, Fms-like tyrosine kinase 3 internal tandem duplication; Treg, Regulatory T cell; iTreg, Inducible Regulatory T cells; CD8+ T Cell, Cluster of Differentiation 8 Positive T Cell; CD4+ T Cell, Cluster of Differentiation 4 Positive T Cell; TGF: Transforming Growth Factor; IL: Interleukin; IFN, Interferon; TNF: Tumor Necrosis Factor; TCR, T Cell Receptor; LAG, Lymphocyte activation gene; TIGIT, T cell immunoreceptor with Ig and ITIM domains; PD-1, Programmed cell death protein 1; TIM, T-cell immunoglobulin and mucin-domain containing; CLIP, Class II-associated invariant chain peptide; Gal-9, Galectin-9; IDO, Indoleamine 2,3-dioxygenase; *CCN3*, Cysteine-rich angiogenic protein 3; *SOCS2*, Suppressor of Cytokine Signaling 2.

In addition, immune evasion is also achieved through the following mechanisms: upregulation of co-expressed immunosuppressive molecules on CD8+ T cells (38), and the elevated expression of immune checkpoint receptors like TIM-3 and LAG-3 (29). Although the proportion of CD8+ T cells is increased in these patients, they typically exhibit a TIGIT+PD-1+DNAM-1− phenotype (38). The co-expression of these immunosuppressive molecules is associated with poorer prognosis (39). However, although no significant differences in the expression of TIGIT and PD-1 were observed between the *FLT3-ITD* mutant and wild-type groups when analyzed in the overall T cell population in some samples (15), it is important to note that the immunosuppressive effects are primarily determined by the expression of immune checkpoint receptors on T cells that specifically recognize leukemia antigens (40). Additionally, following mutation, the *FLT3* receptor remains aberrantly activated, and in combination with the effect of *FLT3* ligand, this leads to elevated expression of TIM-3 in T cells (29). The autocrine or paracrine signaling pathways of TIM-3 promote leukemia cell proliferation and anti-apoptotic activity, while also suppressing the function of distant immune cells (29). TIM-3 expression is accompanied by galectin-9 secretion, which inhibits T cell activity. TIM-3 transcript levels correlate with CLIP levels (29), suggesting that immune evasion mechanisms are often co-activated. Moreover, In the context of *FLT3-ITD* mutation, the expression of the immune checkpoint LAG-3 is significantly increased in T cell subsets (27). LAG-3 impairs T cell receptor (TCR)-mediated signaling, thereby affecting the proliferation and function of T_effs_ cells. High LAG-3 expression is associated with shorter OS and disease-free survival in AML patients (27, 39) (**Figure 1**).

#### 3.1.2 DC expansion and dysfunction in *FLT3-ITD* mutant AML

**Figure 2:**
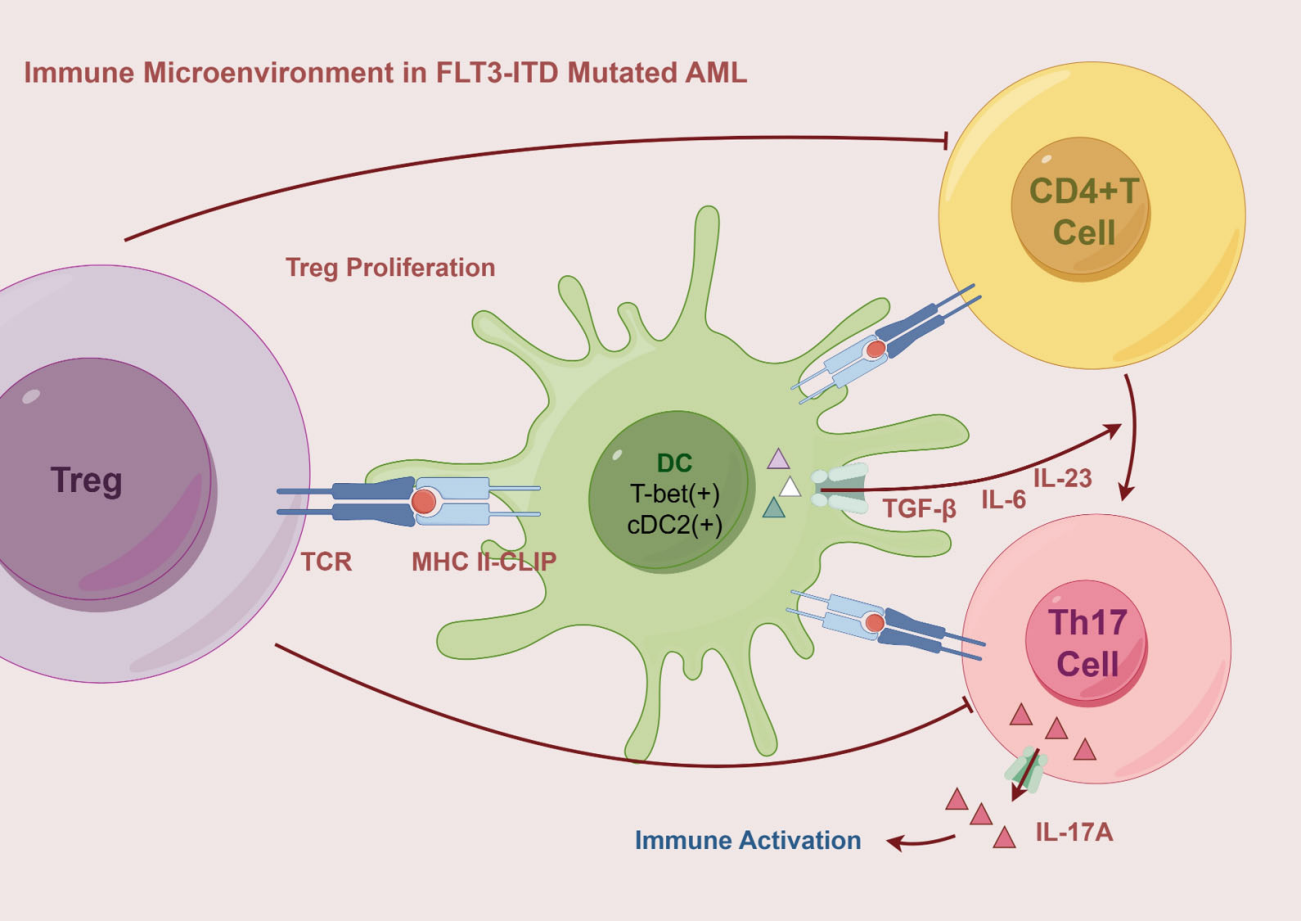
The immune microenvironment in *FLT3-ITD* mutated AML is characterized by the expansion of DCs, particularly the cDC2 subset, which exhibits a T-bet-positive phenotype and persistent CLIP exposure, leading to impaired antigen presentation. Through HLA II-CLIP, DCs promote Treg proliferation and secrete TGF-β, IL-6, and IL-23, driving the polarization of CD4+ T cells into Th17 cells. These Th17 cells produce IL-17A, which contributes to immune activation and inflammatory responses. AML, Acute Myeloid Leukemia; MHC, Major Histocompatibility Complex Class; T-bet, T-box transcription factor expressed in T cells; DC, Dendritic cell; cDC2, Conventional Dendritic Cell Subtype 2; Th17, T-helper 17 Cell.

Compared to wild-type AML patients, those with *FLT3-ITD* mutations exhibit a significant expansion of DCs, particularly common DC progenitors and precursor DCs. In mouse models, the effect of *FLT3-ITD* on DCs is allele dose-dependent; the more copies of the mutation present, the greater the expansion of DCs. This expansion promotes the proliferation of T_regs_, a phenomenon that becomes especially pronounced in the BM as the mutation burden increases (36, 41). Concurrently, DCs undergo abnormal phenotypic changes. The frequency of XCR1/cDC1 double-negative cDCs is markedly elevated, and these cells display impaired antigen presentation capabilities (16). In *FLT3-ITD* mutant patients, CLIP on the surface of cDCs remains bound to and exposed on HLA molecules. CLIP is an invariant chain polypeptide essential for HLA class II antigen presentation and can also be cross-presented on HLA class I molecules. Persistent exposure to CLIP disrupts T cell activation and is associated with poorer prognosis (29). Additionally, compared to healthy mice, the cDC phenotype in *FLT3-ITD* mutant mice is skewed toward T-bet-expressing cDC2. Under the influence of specific cytokines, these cDC2 cells effectively polarize naive CD4+ T cells into Th17 cells, leading to increased production of IL-17A. This Th17 subpopulation has been linked to unfavorable prognosis in AML (16) (**Figure 2**). Meanwhile, the immune evasion mechanisms are further complicated by alterations in TNF secretion by macrophages. The secretion of TNF by macrophages is decreased in patients with *FLT3-ITD* mutations (42), but TNF exerts a dual effect on tumor cells. Under normal conditions, TNF is involved in regulating T lymphocyte-mediated homeostasis and anti-tumor responses, thereby improving CR rates and extending event-free survival. However, studies have shown that lower levels of TNF may promote the death of tumor-infiltrating T cells, enhance tumor cell differentiation, and facilitate the migration of myeloid cells, thereby accelerating leukemia progression (1). Therefore, the role of TNF in AML is complex, and changes in its levels may have varying effects on disease progression.

#### 3.1.3 Macrophage polarization and immune modulation in *FLT3-ITD* mutant AML

The frequency of TIGIT+ M2 macrophages is elevated in AML patients with the *FLT3-ITD* mutation. The high infiltration and expression of TIGIT in M2 macrophages are significantly associated with poor prognosis in AML (14). In AML cells harboring both *FLT3-ITD* mutations and the *KMT2A*::*MLLT3* fusion, the genes *CCN3* and *SOCS2* become activated (43). Activation of *CCN3* recruits macrophages and promotes their differentiation into the M2 phenotype, downregulates the expression of CD36 and SRA1, and consequently reduces phagocytic function (44). Additionally, as a target gene of FoxO1, *CCN3* activation enhances the functionality of T_regs_ (45). *SOCS2* plays a multifaceted role by not only inhibiting the expression of pro-inflammatory cytokines and the development of T helper 2 (Th2) cells in DCs but also promoting the polarization of CD4+ T cells into inducible regulatory T cells (iTregs) (46) (**Figure 1**). It maintains the stable expression of *Foxp3* in iTregs by inhibiting the IL-4 signaling pathway, thereby sustaining the anti-inflammatory phenotype and cellular stability of iTregs (47). Moreover, excessive activation of *SOCS2* leads to the inhibition of IL-8 secretion and upregulation of RANTES expression, both of which are associated with poor prognosis (48). These findings align with observations in AML, where elevated levels of *SOCS2* correlate with reduced OS (43).

#### 3.1.4 Altered NK cell proportions and function in *FLT3-ITD* mutant AML

In addition, the proportion of NK cells in AML patients with *FLT3-ITD* mutations was significantly elevated, and the copy number, ITD length, and mutant allele frequency of *FLT3-ITD* mutations were positively correlated with the proportion of NK cells. However, patients with a high proportion of NK cells at the initial stage of AML tend to have a poor prognosis. This may be due to a reduced number of mature NK cells and their limited cytotoxic function (37). Furthermore, the expression of inhibitory receptors is generally increased across all NK cell subsets, although it exhibits heterogeneity (8). In a study by Cianga’s team analyzing BM samples from eight AML patients, the proportion of overmature NK cells in patients with *FLT3* mutations was significantly increased (9). Conversely, in another study by the same team examining PB from 20 newly diagnosed AML patients, those with *FLT3* mutations exhibited an extremely low proportion of NK cells and markedly abnormal expression levels of the inhibitory receptor CD159a (8). These discrepancies may be attributed to differences in patient cohorts, variations between the BM and PB environments, and the regulatory influence of the TME on NK cell development.

#### 3.1.5 Dual role of MAIT cells in tumor surveillance and immune evasion in *FLT3-ITD* mutant AML

AML patients with *FLT3-ITD* mutations have increased numbers of mucosal-associated invariant T (MAIT) cells, which predominantly exhibit effector memory or terminally differentiated phenotypes (35), indicating high activation but also signs of aging and exhaustion, characterized by upregulated PD-1 and downregulated CD161 expression (49). Moreover, MAIT cell function is compromised, with reduced Th1-type cytokine production (IFN-γ and TNF-α) and increased secretion of Th17-type cytokines (IL-17A and IL-8), granzyme B, and perforin (49). We speculate that in AML patients, despite reduced Th1-type cytokine production, MAIT cells may exert anti-tumor effects primarily through granzyme B and perforin-mediated degranulation and cytokine activation, as indicated by the increased secretion of Th17-type cytokines and cytotoxic molecules. These findings suggest that MAIT cells have a dual role in AML, functioning both as anti-tumor agents and potentially as tumor promoters. However, the changes in PD-1 and CD161 expression and the secretion of immune effector molecules in the context of *FLT3-ITD* mutations require further investigation.

### 3.2 *NPM1* mutation

*NPM1* mutations occur in approximately 20% to 30% of adult AML patients. Over 80% of these mutations are type A, characterized by a frameshift insertion at the fourth nucleotide position. This mutation alters the last 11 amino acids at the C-terminus of the *NPM1* protein, resulting in its abnormal retention in the cytoplasm, referred to as *NPM1*c (50–52).

#### 3.2.1 *NPM1* mutation-specific T cell responses in AML

**Figure 3:**
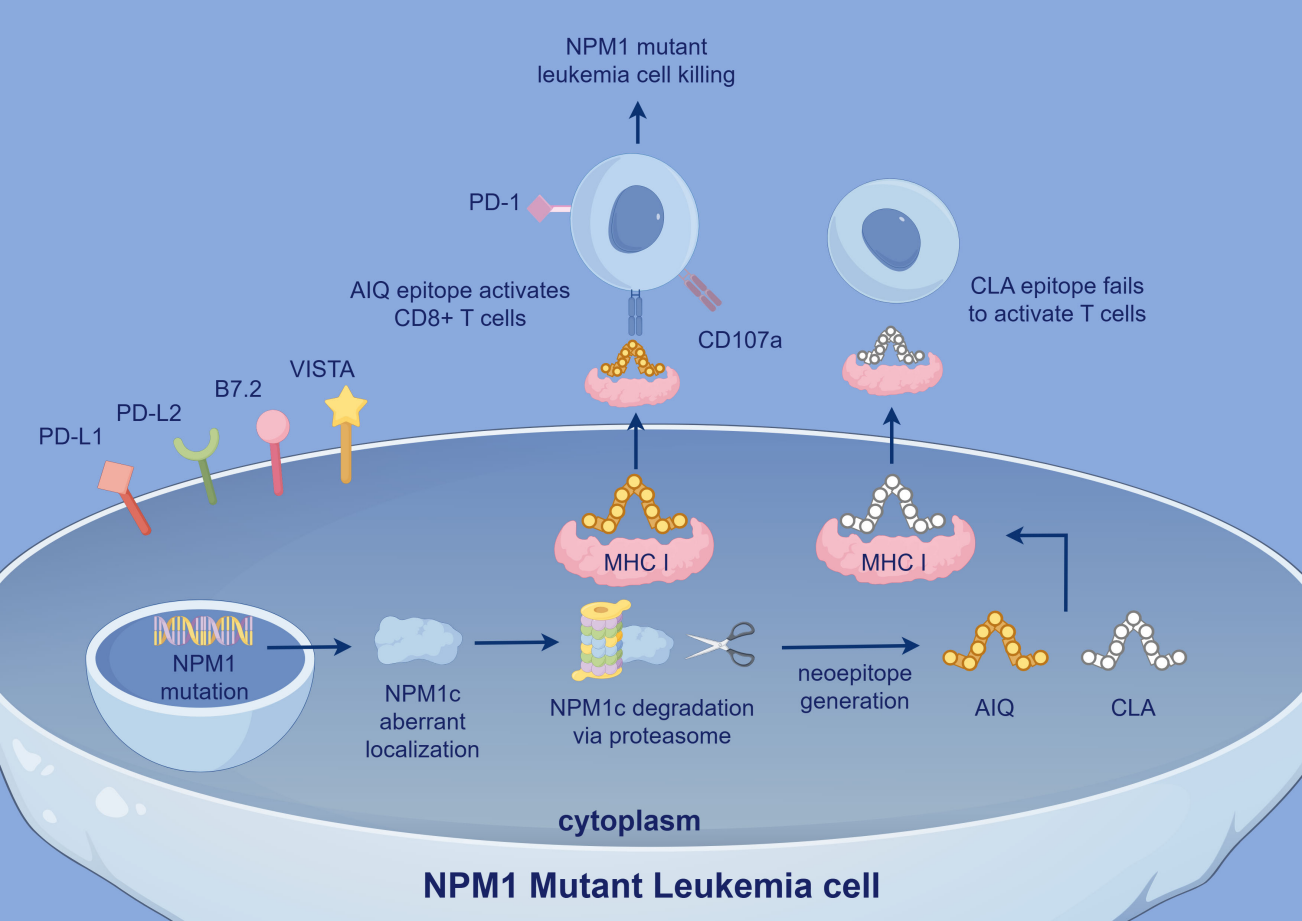
In *NPM1* mutant leukemia cells, aberrant *NPM1*c localization leads to the generation of neoepitopes, including AIQ and CLA. The AIQ epitope activates CD8+ T cells via MHC I presentation and induces leukemia cell killing, with CD107a expression indicating T cell activation. In contrast, the CLA epitope fails to activate T cells. Immune checkpoint molecules such as PD-1, VISTA, and PD-L1 also contribute to immune evasion. *NPM1*, Nucleophosmin 1; *NPM1*c, Cytoplasmic Nucleophosmin 1 (mutated form of *NPM1* abnormally localized to cytoplasm); PD-L2: Programmed Death-Ligand 2; VISTA, V-domain Ig Suppressor of T cell Activation; AIQ, AIQDLCLAV; CLA, CLAVEEVSL.

The abnormal cytoplasmic localization of *NPM1*c leads to the generation of novel neoepitopes, including AIQDLCLAV (AIQ) (5) and CLAVEEVSL (CLA) (53), which can be recognized by specific TCRs. The AIQ epitope, presented by HLA-A2, can bind to specific TCRs, and T cells engineered to express these TCRs effectively kill *NPM1*c+HLA-A2+ AML cells. AML patients with *NPM1*c+ who exhibit AIQ-specific CD8+ T cell responses have significantly longer survival (5). In contrast, the CLA epitope does not elicit a significant T cell response (53), suggesting that CLA may be suppressed by the TME *in vivo*. *NPM1* mutation-specific CD8+ T cells can directly lyse leukemia cells harboring *NPM1* mutations, whereas CD4+ T cells support CD8+ T cell function and induce HLA class II-mediated anti-tumor cytotoxic responses. These specific T cells predominantly express CD107a (54), indicating their activated state and involvement in cytotoxic activity (**Figure 3**). Notably, specific T cell responses can still be detected after morphological CR in AML patients. This suggests that even when leukemic cells are substantially reduced, these T cells continue to eliminate minimal residual disease (MRD), thereby maintaining long-term CR and reducing relapse risk. A decrease in specific T cells is associated with disease relapse, and patients exhibiting these T cell responses have longer OS (54), further indicating a strong correlation with better prognosis. However, it remains to be investigated whether the frequency and intensity of *NPM1* mutation-specific T cell responses vary based on patients’ molecular characteristics.

#### 3.2.2 Immune checkpoint regulation and inhibitory ligands in *NPM1*-mutated AML

AML cells can still evade immune surveillance through multiple mechanisms. In particular, under the influence of neoantigenic epitopes produced by *NPM1* mutations, leukemic cells predominantly express inhibitory B7 family ligands (such as PD-L1, PD-L2, B7.2, and VISTA) (51, 55). Elevated PD-L1 expression significantly enhances the immunosuppression of *NPM1* mutation-specific CD8+ T cells (56). This high level of PD-L1 is strongly associated with poorer patient outcomes, in part due to the expansion of T_regs_ (57), especially in patients with concurrent *FLT3-ITD* mutations (51, 54). Although some studies suggest that *NPM1* is essential for PD-L1 expression and that *NPM1* mutations may slightly reduce PD-L1 expression, these differences are not statistically significant (58), and the underlying mechanisms remain to be elucidated. Furthermore, In patients with *NPM1*-mutated AML, TIM-3 transcript levels are also significantly reduced (58). TIM-3 plays a complex role in immune function, correlating with T cell exhaustion while also enhancing NK cell cytotoxicity. Some studies indicate that low TIM-3 expression may be associated with favorable prognosis in *NPM1*-mutated AML, whereas higher TIM-3 expression is linked to significantly lower CR and survival rates at one-year follow-up (58). These findings suggest that TIM-3 may serve as a potential indicator of poor prognosis. Notably, patients with a greater number of TIM-3+ NK cells exhibit better prognoses (59), emphasizing the dual nature of TIM-3 in AML. Overexpression of the pro-inflammatory mediator LTB4R is positively correlated with the expression of inhibitory immune checkpoint molecules such as PD-1 and TIM-3, while showing a negative correlation with immune effector cell populations (60). Although the mechanistic link remains unclear, this suggests that LTB4R may contribute to the immunosuppressive landscape in *NPM1*-mutated AML. In addition, *NPM1* mutations have been shown to enhance the expression of CD47, a key ‘don’t eat me’ signal, which interacts with SIRPα on macrophages to inhibit phagocytosis. This mechanism further protects leukemic cells from immune clearance by the innate immune system (58). Although activating signals such as ULBP1 can stimulate NK and T cell responses through engagement with the NKG2D receptor, their immunostimulatory effects may be attenuated by the concurrent upregulation of immune checkpoint molecules, further enabling immune evasion in *NPM1*-mutated AML (51).

#### 3.2.3 *NPM1* mutation-mediated modulation of HLA expression and antigen presentation in AML

The antigen presentation process is also inhibited. In the absence of *DNMT3A* mutations, *NPM1* mutations lead to the downregulation of the *CIITA* gene (51), thereby inhibiting the expression of CLIP protein and HLA molecules, which helps leukemia cells evade recognition by CD8+ T cells. However, AML cells partially retain HLA expression, and cells with low HLA expression do not exhibit higher NK cell lysis rates (58), suggesting that leukemia cells may balance NK and T cell attacks in immunoediting by regulating HLA levels. Furthermore, studies have found that the frequency of specific HLA-I alleles in patients with *NPM1* mutations is significantly lower than that in healthy controls and *NPM1* wild-type AML patients (54). This suggests that HLA alleles capable of effectively presenting *NPM1* peptides may reduce the risk of developing *NPM1*-mutated AML. Even among patients carrying such alleles who develop the disease, specific immune responses may contribute to disease remission. *DNMT3A* mutations can weaken the effect of *NPM1* mutations on HLA expression. Interestingly, in samples with high HLA-DR expression, *NPM1* mutations are associated with higher CLIP levels, indicating a complex regulation of antigen presentation and immune responses (58).

#### 3.2.4 Metabolic and costimulatory dysregulation in *NPM1*-mutated AML

In addition to modulating classical immune checkpoint pathways, *NPM1* mutations suppress immune function through a range of noncanonical mechanisms. One such mechanism involves the regulation of small extracellular vesicle-mediated signaling. The *NPM1*c/CTCF/PABPC1 signaling axis controls the secretion of miR-19a-3p via small extracellular vesicles, which are subsequently internalized by CD8+ T cells. This process inhibits the expression of creatine transporters, prevents creatine uptake, reduces ATP production, and consequently impairs the immune function of CD8+ T cells (53). Moreover, *NPM1* mutations significantly upregulate *SPINK2* expression and downregulate *ALCAM*, both of which contribute to impaired T cell activation (61).

### 3.3 *DNMT3A* mutation

Approximately 25% of AML patients harbor mutations in the *DNMT3A* gene, with the R882H variant being the most prevalent. This mutation reduces *DNMT3A*’s methyltransferase activity and is associated with genome-wide hypomethylation. Some studies suggest that this hypomethylation represents an early event initiated by the mutation, whereas *DNMT3A*-dependent CpG island hypermethylation may emerge during AML progression (62). In addition, *DNMT3A* mutations are frequently associated with increased chemotherapy resistance (63–65). Although study results have varied (66), such discrepancies may be attributed to differences in patient population characteristics.

#### 3.3.1 T cell subset imbalance and functional consequences in *DNMT3A*-mutated AML

*DNMT3A* plays a pivotal role in shaping immune cell fate, particularly by maintaining the differentiation stability of CD4+ T cells and restricting both the formation of long-term memory CD8+ T cells and the pool of memory precursor effector cells (28, 67). In the context of AML, *DNMT3A* mutations have been associated with notable alterations in T cell infiltration and subset composition. In AML patients, *DNMT3A* mutations are associated with increased T cell infiltration; however, the distribution of T cell subsets is aberrant. Specifically, in patients with wild-type *DNMT3A*, T cells tend to undergo terminal differentiation, resulting in a reduced proportion of memory T cells (28). In contrast, patients with *DNMT3A* mutations exhibit a reduction in CD8+ T_n_ and CD4+ T_em_ in the BM, accompanied by an increase in CD4+ central memory T cells. Clinical studies have demonstrated that using donors with a higher proportion of CD8+ T_n_ for lymphocyte infusion can contribute to long-term remission in AML patients (28). Notably, similar to other mutations affecting DNA methylation regulators such as *NPM1*, *IDH2*, and *CEBPA*, *DNMT3A* mutations have also been shown to upregulate tumor-specific antigens, which in turn can activate antigen-specific clonal T cell responses (68). Conversely, lower ratios of CD8+ T_n_ and CD4+ T_em_ are associated with adverse genetic risks and poorer relapse-free survival and event-free survival (28). The specific impact of T_em_ cells on prognosis remains controversial. Some studies, such as those by Ling Xu and Adam J. Lamble, have found that an increased proportion of T_em_ cells is associated with enhanced T cell proliferation and higher CR rates. However, Maddalena Noviello’s team reported that the proportion of T_em_ cells also increases in relapsed AML patients (28). Moreover, *DNMT3A* mutations are linked to a higher risk of acute graft-versus-host disease following allogeneic hematopoietic stem cell transplantation, primarily by promoting CD4+ T cell polarization toward a Th1 phenotype and enhancing IFN-γ production (67). These findings collectively highlight the profound impact of *DNMT3A* mutations on T cell differentiation, function, and clinical outcomes in AML.

#### 3.3.2 Immunosuppressive mechanisms and innate immune impairment driven by *DNMT3A* mutations

Beyond modulating adaptive immunity, *DNMT3A* mutations also disrupt innate immune signaling and promote an immunosuppressive TME. One such mechanism involves the hypomethylation-induced upregulation of *miR-196b*, which directly inhibits the Toll-like receptors (TLR) 7/8 signaling pathway, thereby weakening the immune response. In normal immune cells, TLR7 activates type I IFN and cytokines through the MyD88 pathway, indirectly activating Stat1 signaling to enhance the activity of Th1 cells and monocytes while promoting DC differentiation (69). Consequently, inhibition of TLR7/8 may lead to a diminished overall immune response. *DNMT3A* mutations are also frequently accompanied by increased infiltration of T_regs_ (70). Previous studies have shown that T_regs_ are highly adaptable to different tissue environments, and *DNMT3A*-dependent *de novo* DNA methylation facilitates this adaptability by establishing tissue-specific epigenetic memory, thereby refining and modulating their functions. However, in AML, *DNMT3A* mutations may impair methyltransferase activity, making it difficult for T_regs_ to adapt to various environments and thereby affecting their immune regulatory functions (71). Additionally, *DNMT3A*-dependent *de novo* DNA methylation is essential for silencing *Foxp3* transcription. Mutations in *DNMT3A* may impair the effective silencing of *Foxp3* transcription in T_regs_, allowing them to continuously maintain their immunosuppressive functions, which may further promote immune escape in AML (72). In addition to T_reg_-mediated suppression and impaired TLR signaling, *DNMT3A*-mutated AML cells also exhibit elevated levels of immunosuppressive cytokines such as IL-10 and TGF-β (62). Furthermore, the concurrent upregulation of immune checkpoint molecules including PD-L1, CLIP, and TIM-3 indicates a coordinated activation of multiple immune evasion pathways, collectively contributing to a highly suppressive TME and poor prognosis (58).

#### 3.3.3 Myeloid reprogramming and TAM polarization in *DNMT3A*-mutated AML

In addition to its effects on lymphoid immunity, *DNMT3A* mutation significantly alters the myeloid compartment. AML cells harboring *DNMT3A* mutations have been shown to possess an enhanced capacity to chemoattract monocytes, thereby modifying the TME to favor immune suppression. These AML cells inhibit the activity of the AP-1 binding site, leading to the downregulation of pro-inflammatory cytokines such as MIP-1α, MIP-1β, and IL-1β (62). The suppression of these key mediators impairs M1 macrophage polarization and diminishes their cytotoxic functions against tumor cells. *In vivo* studies using murine models have demonstrated a marked increase in the proportion of M2-polarized tumor-associated macrophages (TAMs) in the presence of *DNMT3A* mutations. These M2 macrophages express high levels of CD163 and CD206 and secrete chemokines such as CCL17, CCL22, and CCL24, which recruit Th2 cells to the leukemia microenvironment (62). The accumulation of M2 TAMs and Th2 cells creates an anti-inflammatory milieu that correlates strongly with reduced patient survival. Furthermore, *DNMT3A*-mutated AML cells exhibit resistance to macrophage-mediated killing and can differentiate into monocyte-like cells with immunosuppressive properties, further contributing to the inhibition of effective anti-leukemic T cell responses (62). These findings underscore a critical role for *DNMT3A* mutations in reprogramming the myeloid landscape, skewing macrophage polarization toward an immunosuppressive phenotype, and establishing a tumor-permissive microenvironment.

### 3.4 *TP53* mutation

In AML, the detection rate of *TP53* gene mutations is approximately 5-10%. However, this rate is significantly higher in treatment-related AML and in elderly patients with complex karyotypes, reaching as high as 70-80%. *TP53* mutations are recognized as an independent prognostic factor for poor outcomes in AML patient (73).

#### 3.4.1 T cell dysfunction, exhaustion, and T_regs_ expansion in *TP53*-mutated AML

**Figure 4:**
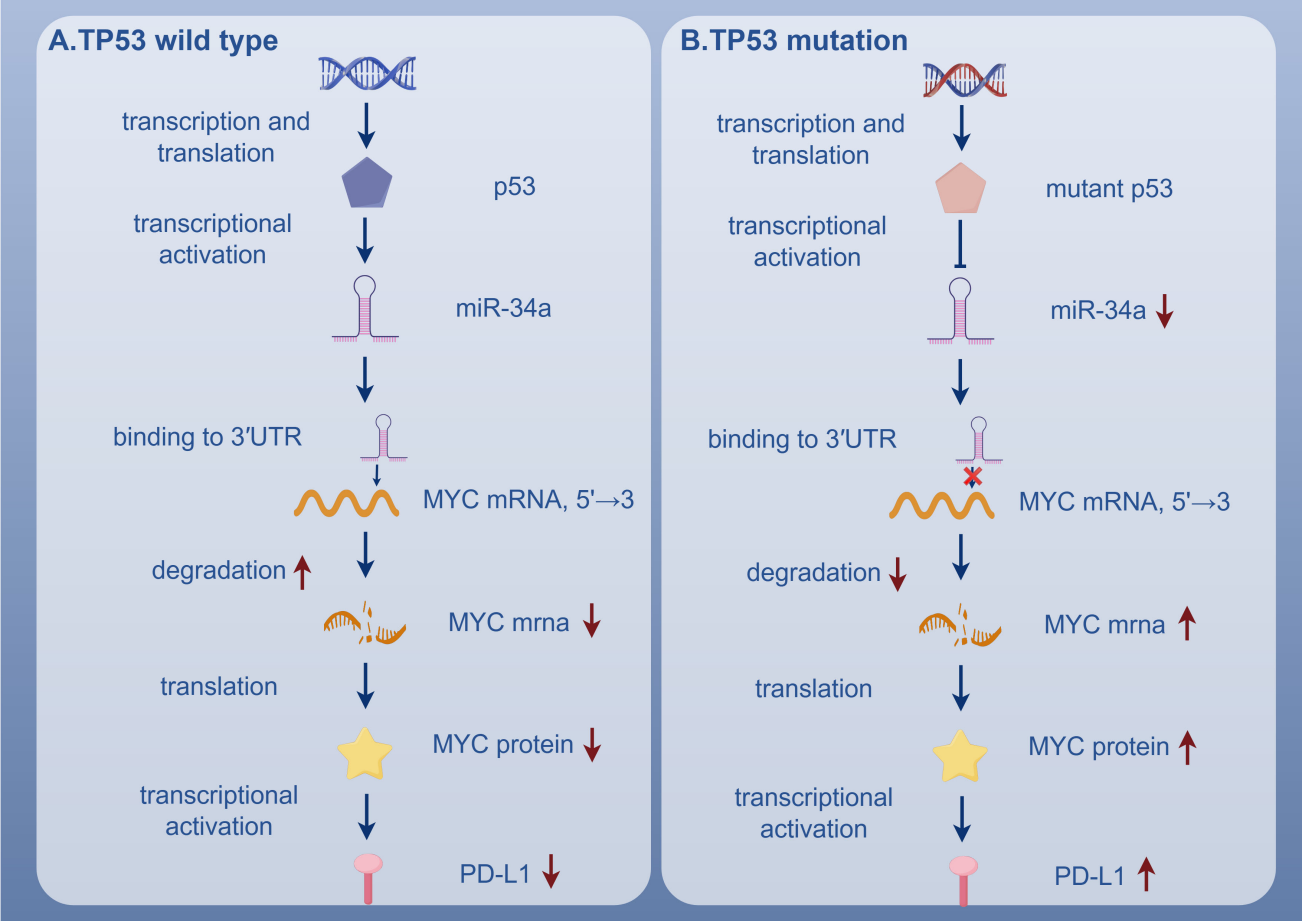
In *TP53* wild-type AML, p53 promotes miR-34a expression, leading to MYC mRNA degradation and suppression of PD-L1 expression. In contrast, *TP53*-mutated AML shows reduced miR-34a, resulting in MYC upregulation and PD-L1 overexpression. *TP53*, Tumor Suppressor P53 Gene; p53, Tumor Protein 53; miR-34a, MicroRNA-34a; 3'UTR, 3' Untranslated Region; MYC Mrna, Myelocytomatosis Oncogene Messenger RNA; PD-L1, Programmed Death-Ligand 1.

*TP53* mutations in AML lead to substantial alterations in the function and composition of T cells. Despite the increased infiltration of T cells, these cells exhibit signs of exhaustion and impaired functionality (71). Specifically, the expression of activation markers such as CD25, HLA-DR, and CD127 is low (74), while the immune checkpoint receptor CTLA-4 is upregulated (75). Moreover, the expression of cytotoxic molecules such as perforin and granzyme B is diminished (76), and the secretion of Th1 cytokines is significantly reduced (77). The T cell subsets are also significantly altered. Unsupervised clustering analysis revealed that CD8+ T cells predominantly exhibit an ICOS+/4-1BB+/PD-1+ phenotype (78), suggesting a dysfunctional, exhausted state. In contrast, Th cells predominantly express ICOS (6), which, despite being a costimulatory molecule, contributes to immune evasion in this context. The increase in PD-1+ cytotoxic T lymphocytes and PD-L1+ BM blasts further supports this hypothesis, as PD-1 signaling has been shown to inhibit T cell function and induce exhaustion (79). The upregulation of PD-L1 is closely associated with the downregulation of miR-34a and the overexpression of the *MYC* gene. Under normal conditions, wild-type p53 induces the transcription of miR-34a, which targets *MYC* mRNA and promotes its degradation, thereby negatively regulating *MYC* expression. However, in *TP53*-mutated AML, miR-34a expression is significantly reduced, leading to the upregulation of *MYC* and the induction of PD-L1 expression. The downregulation of miR-34a weakens its binding to the 3′ untranslated region of PD-L1 mRNA, reducing the inhibition of PD-L1 expression and thereby promoting T cell exhaustion (6, 78) (**Figure 4**). Transcriptional analysis revealed that, compared to healthy controls, cytotoxic T lymphocytes from *TP53*-mutated AML patients exhibited upregulation of inhibitory molecules (such as CD244, CD160, LILRB1, CD300A, and PVRIG) and downregulation of stimulatory molecules (such as CD40LG, CD28, TNFSF8, TMIGD2, and TNFRSF25). These alterations were not significant in other AML molecular subtypes (79).

In contrast, T_regs_ demonstrated metabolic adaptations and proliferative advantages. Although OX40+ T_regs_ are markedly reduced in the BM, the highly immunosuppressive ICOShigh/PD-1neg T_regs_ are significantly expanded (6). These T_regs_ exhibit enhanced proliferative capacity and are implicated in the suppression of anti-tumor immunity, which has been identified as an independent predictor of poor OS (78). The IL-2/STAT5 signaling axis further promotes T_reg_ differentiation and stabilization (74, 80), with elevated *FOXP3* expression (74) and increased secretion of immunosuppressive cytokines such as TGF-β and IL-10 (76). Together, these findings illustrate a significant alteration in T cell functionality, with exhaustion of T_effs_ and expansion of suppressive T_regs_, contributing to the immune escape observed in *TP53*-mutated AML.

#### 3.4.2 Dysregulation of innate immune pathways and pro-inflammatory microenvironment in *TP53*-mutated AML

*TP53* mutations in AML significantly affect innate immune and leads to the formation of a pro-inflammatory microenvironment. One important mechanism is the downregulation of HLA molecules, which impairs antigen presentation, thus preventing the immune system from effectively recognizing and eliminating leukemia cells (74, 76). In addition, despite promoting significant infiltration of TAMs (80), the overexpression of CD47 on leukemia stem cells interacts with the SIRPα receptor on TAMs, inhibiting their phagocytic activity (81). Additionally, *TP53* mutations lead to the upregulation of JAK/STAT, PI3K-Akt, and NF-κB signaling pathways, which are associated with increased production of pro-inflammatory cytokines such as CXCL1, CXCL2, CXCL8/IL-8, and IFN-induced products like CCL2, IL33, and IL6 (75). This results in the formation of a pro-inflammatory microenvironment, with IFN-γ playing a dominant role, which has been linked to poor responses to induction chemotherapy. Moreover, *TP53* mutations inhibit IRF3 transcriptional activity and affect IFN expression through two mechanisms (1): by binding to TBK1, preventing the formation of the STING-TBK1-IRF3 complex (82), and (2) by inducing the overexpression of *PLK4*, which further inhibits the activation of the cGAS-STING-TBK1-IRF3 pathway (83).

### 3.5 *IDH1/2* mutation

Mutations in *IDH1/2* are present in approximately 15-20% of AML patients. Gain-of-function mutations in these enzymes can lead to a blockade in hematopoietic cell differentiation and promote leukemic transformation (84). In AML, mutant *IDH1/2* enzymes convert α-ketoglutarate into the oncometabolite 2-hydroxyglutarate (2-HG), which accumulates in tumor tissues and patient serum, thereby suppressing immune function. 2-HG limits the secretion of CXCL10 by tumor cells, reducing T cell recruitment to tumor sites (85). Additionally, it inhibits the differentiation of monocytes into DCs, decreases the expression of HLA-DQ and HLA-DR on DCs, induces a tolerant phenotype, and suppresses the upregulation of DC markers such as CD1a and DC-SIGN. This results in reduced IL-12 and increased IL-10 secretion, thereby weakening the ability of DCs to stimulate T cells. AML cells harboring *IDH* mutations also exhibit decreased HLA-DP expression and demonstrate increased resistance to lysis by HLA-DP-specific T cells (86). However, Sunthankar KI reported that AML cells with the *IDH2 R140Q* mutation show increased HLA-DR expression and are capable of inducing T cell immune responses (84). Furthermore, once absorbed by immune cells, 2-HG inhibits histone and DNA demethylation in mouse CD8+ T cells, activates HIF-1α, and impairs T cell proliferation and effector functions. In human T cells, 2-HG destabilizes HIF-1α, promotes oxidative phosphorylation, enhances differentiation into CD4+CD25+FOXP3+ T_regs_, and inhibits Th17 cell differentiation. Additionally, 2-HG is transported into T cells via SLC13A3, where it interferes with NFATC1 signaling, limits T cell proliferation and function, and induces ATP depletion by inhibiting oxidative phosphorylation (85), thereby further enhancing immunosuppression. In stromal cells, 2-HG upregulates NF-κB and enhances the NF-κB phosphorylation response of *IDH2* mutant cells under IL-1β stimulation, leading to abnormal cytokine secretion (84). Most of these mechanisms facilitate AML progression and tumor immune evasion. However, the immune microenvironment also contains anti-tumor effector cells. For instance, *IDH1/2* mutations can induce a significant increase in MAIT cells (33) and CD4+ T_effs_ (26).

### 3.6 *NRAS* mutation

*NRAS* mutations are found in approximately 15-20% of AML patient (87). Multiple studies have shown that *NRAS* mutations alone have no significant impact on prognosis, but are associated with higher survival rates after adjusting for age and other factors (88). This suggests that *NRAS* mutations may predict a better prognosis under certain conditions, but further verification is needed. *NRAS* mutations show strong antigen presentation potential. Specifically, in the *NRAS^G12D* mutant AML mouse model, hematopoietic stem/progenitor cells upregulated the expression of MHC class molecules, driving a potent anti-leukemia response. When the *RUNX1-RUNX1T1* fusion gene is present, the expression levels of H2-Db and H2-Kb of MHC class I molecules are also significantly increased (89). Compared with the normal control group, the proportion of CD4+ T cells in the mutant group of mice was significantly reduced and the proportion of CD8+ T cells was significantly increased, indicating that the adaptive immune response was activated. However, expression levels of the *PD-1* gene were increased in T cells, suggesting that *NRAS^G12D* AML cells evade immune surveillance by activating and depleting T cells. When T cells express inhibitory receptors and enter a state of exhaustion, disease is more likely to develop. *NRAS*^G12D AML, which is highly immunogenic, exhibits immunoediting in mice and upregulates PD-L1 expression. In mutation models, anti-PD-1 treatment has limited effect on relieving T cell suppression and the recovery of anti-leukemia immune responses is also limited, suggesting that *NRAS* mutant AML evades immune system surveillance through multiple mechanisms (89). When *NRAS* and *ASXL1* are double mutated, AML cells also hyperactivate the MEK/ERK/AP-1 signaling pathway, leading to the upregulation of AP-1-related genes and inhibitory immune checkpoint ligands PD-L2, CD80, CD86, and CD155. This further inhibits the anti-leukemia activity of CD8+ T cells, NK cells and γδ T cells (25).

In summary, various driver gene mutations in AML regulate the immune microenvironment through multiple mechanisms (**Supplementary Table 1**). These mutations can either promote specific immune responses or lead to immune escape and immunosuppression, thereby influencing disease progression and prognosis. A thorough investigation of the relationship between these gene mutations and immune responses will enhance our understanding of the dynamic changes within the AML TME. This understanding provides a theoretical foundation for elucidating the mechanisms of immune escape and developing precise treatment strategies for the disease.

## 4 Immune microenvironment and genetic risk stratification

The TME comprises a diverse array of cellular components (90) and plays a well-established role in supporting tumor survival and progression across both solid and hematological malignancies (91). In AML, the TME exhibits a dual role: while it fosters leukemogenesis, it may also limit the expansion of malignant clones and contribute to their immune-mediated clearance (92). Within this context, the immune microenvironment of AML is highly complex and heterogeneous. Interactions between leukemic cells and immune components are central to disease initiation, progression, immune escape, and therapeutic resistance.

**Figure 5:**
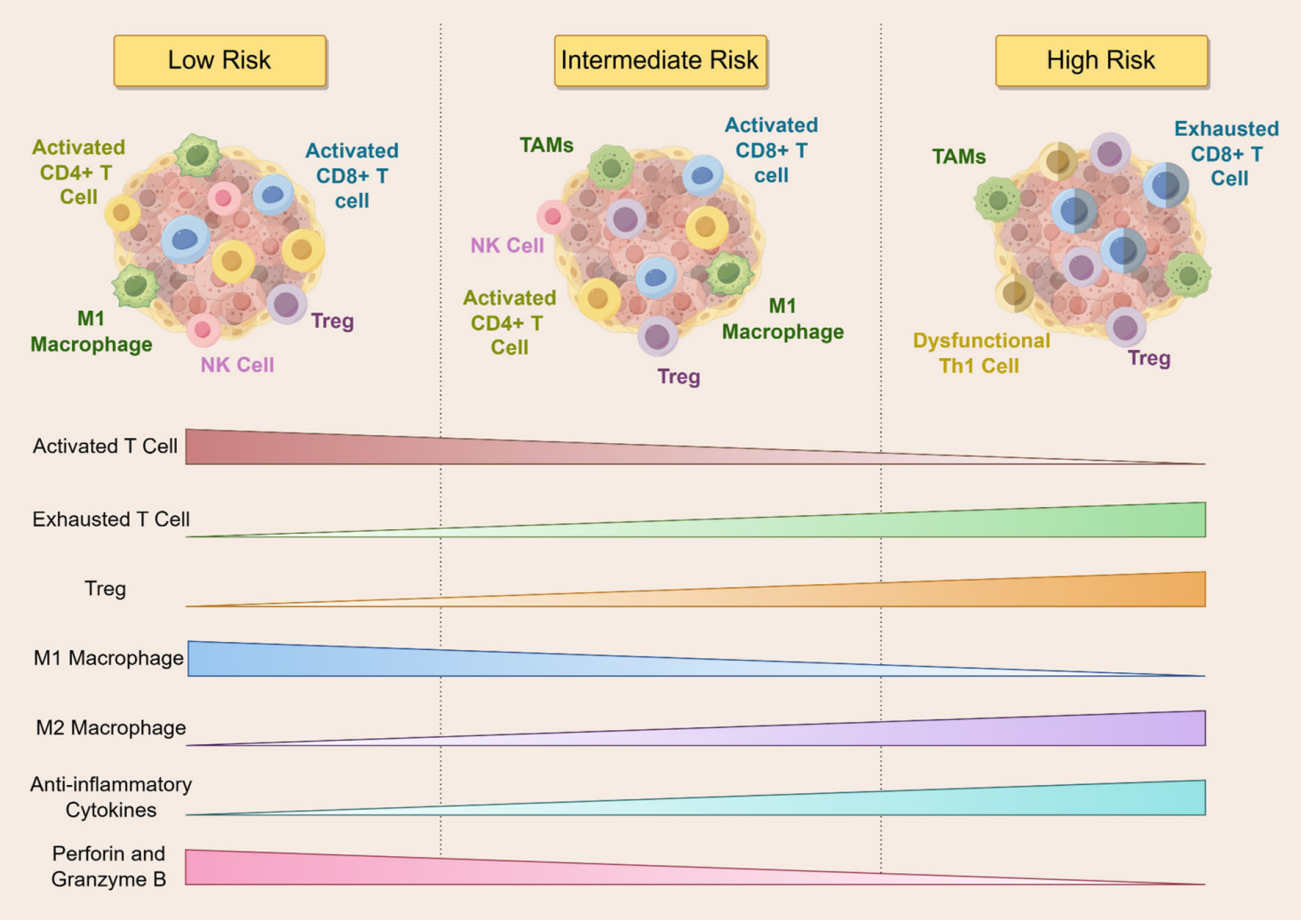
The figure illustrates the dynamics of immune cell infiltration across different risk stratifications in AML patients. As the risk level increases, activated T cells, M1 macrophages, and cytotoxic molecules such as perforin and granzyme B gradually decline, whereas exhausted T cells, Tregs, M2 macrophages, and anti-inflammatory cytokines progressively increase. Low-risk patients exhibit robust anti-tumor immune activity, while high-risk patients are characterized by a markedly immunosuppressive microenvironment. NK Cell, Natural Killer Cell; TAMs, tumor-associated macrophages; Th1, T-helper 1 Cell.

Recent transcriptomic analyses, such as those using TCGA-LAML data, have revealed distinct immune signatures across cytogenetic risk categories (93). To further investigate this, we examined immune microenvironmental profiles based on ELN 2022 (31) and 2024 (32) genetic risk stratifications. According to these guidelines, mutations in *NPM1* and *IDH2 R140* are categorized as low risk; *FLT3-ITD*, *NRAS*, and *DNMT3A* as intermediate risk; and *TP53* and *IDH2 R172* as high risk. Our synthesis suggests that AML patients exhibit markedly different immune landscapes depending on their genetic risk category. Patients with low-risk mutations tend to have immunologically active environments, characterized by elevated effector T cell infiltration, increased pro-inflammatory cytokines, robust antigen presentation, and reduced expression of inhibitory checkpoint molecules (5, 53–55, 94–96). In contrast, high-risk mutation profiles are associated with suppressed T cell activity, impaired antigen presentation, an abundance of T_regs_, and increased levels of immunosuppressive cytokines (6, 71, 74–81). Intermediate-risk groups appear to exhibit a transitional immune state with both pro-inflammatory and immunosuppressive features, reflecting a dynamic equilibrium. These observations are well illustrated in **Figure 5**.

While these trends are compelling, several limitations warrant consideration. Many referenced studies rely on bulk transcriptomic data or immune deconvolution algorithms, which may not accurately capture the spatial and functional heterogeneity of immune cells in the AML microenvironment. Moreover, although immune activation is generally associated with favorable prognosis, the clinical significance of certain immune signatures—particularly in high-risk subtypes—remains controversial. Some studies have reported paradoxical findings, such as activated T cell phenotypes coexisting with immune dysfunction in high-risk groups (97). Additionally, immune infiltration is modulated by variables such as clonal hematopoiesis (98), treatment history (99), and the bone marrow niche (100), complicating its prognostic interpretation. Future studies incorporating single-cell and spatial transcriptomics, functional assays, and longitudinal immune profiling will be essential to disentangle the complex relationships between genetic mutations, immune remodeling, and patient outcomes in AML. While our review attempts to summarize and synthesize the current understanding, we acknowledge the rapidly evolving nature of this field and the need for continued critical evaluation of emerging evidence.

## 5 Future developments: immunotherapy strategies targeting neoepitopes

In recent years, the therapeutic landscape of AML has evolved beyond conventional intensive chemotherapy, driven by the urgent need to address high relapse rates and poor long-term survival (101), particularly in older patients and those with comorbidities (102, 103). Despite initial responses, even MRD-negative patients remain at substantial risk of relapse within three years, with rates approaching 70–80% (104). While targeted therapies such as *FLT3* inhibitors, such as midostaurin and gilteritinib (105–107), and *IDH* inhibitors have improved survival in molecularly defined subgroups (108), their durability is limited. Resistance mechanisms—including secondary kinase domain mutations and bypass signaling activation—commonly emerge, often without significantly altering the immunosuppressive TME (108). Immunotherapy has revolutionized the treatment of several hematologic malignancies, yet its impact in AML has remained modest (109). One of the key challenges is the lack of leukemia-specific antigens that distinguish malignant from normal hematopoietic cells, thereby increasing the risk of off-tumor toxicity (101). Furthermore, AML is characterized by profound immune evasion strategies. As such, most current immunotherapeutic strategies benefit only a subset of patients, and their effectiveness is constrained by the highly suppressive immune milieu.

With advances in genomic profiling, driver mutations in AML have emerged not only as prognostic markers but also as potential sources of neoepitopes. These mutant-derived peptides, absent in healthy cells, can be presented via MHC molecules and recognized by T cells (52, 110), rendering them attractive targets for precision immunotherapy (111). Notably, AML harbors a high frequency of insertion/deletion (indel) mutations, which generate disproportionately more high-affinity neoepitopes compared to single nucleotide variants or gene fusions (101). Among the most extensively studied neoepitopes are those derived from *NPM1* mutations. AIQ-specific CD8+ T cells have shown cytotoxic activity and correlate with improved survival, suggesting potential for adoptive T cell therapy (112, 113). While *NPM1*-derived peptides can stimulate immune responses in preclinical models and relapse settings, tumor-driven HLA loss and immune editing may limit long-term effectiveness. Furthermore, studies remain inconsistent regarding the frequency and robustness of these responses across patient subgroups, highlighting the need for standardized immunomonitoring. Similarly, *FLT3-ITD* mutations, which are present in both leukemic blasts and stem cells, generate neoepitopes such as the YVD/A1 peptide (114). TCR-engineered T cells targeting *FLT3D835Y* have demonstrated specificity and efficacy in preclinical settings (115). However, the heterogeneity in *ITD* insertion sites and lengths raises concerns regarding peptide variability and inconsistent T cell responses across patients (116). Moreover, while the immunogenicity of some FLT3-derived peptides is promising (115), their therapeutic potential has yet to be validated in clinical trials. Neoepitopes from *DNMT3A R882H* and *IDH2 R140Q* mutations have also been identified, capable of binding to HLA-A01:01 and HLA-B07:02 respectively (111). These peptides have been shown to elicit memory T cell responses, although supporting evidence in clinical or *in vivo* contexts remains limited. For example, 2-HG, a metabolite produced by mutant *IDH* enzymes, suppresses T cell activation, complicating efforts to harness these neoepitopes for therapy (117). Likewise, while *TP53* mutations are associated with immunosuppressive TME (118, 119), few studies have successfully identified immunogenic peptides from *TP53* variants with therapeutic applicability.

However, a higher neoepitope burden may not translate into improved immunogenicity or therapeutic responsiveness. In contrast to observations in solid tumors, where high neoantigen load often correlates with better immune activation, AML appears to exhibit the opposite trend: chronic exposure to neoantigens may promote T cell exhaustion and immune tolerance, thereby impairing effective antitumor immunity (101). Additionally, neoantigen heterogeneity and clonal evolution further complicate therapeutic targeting, as subclonal neoepitopes may be poorly presented or lack broad applicability (101). These findings underscore the need for caution when interpreting neoepitope quantity as a surrogate for immunotherapeutic potential. Neoepitope-targeted strategies in AML must be carefully integrated into comprehensive therapeutic approaches that also address the profoundly immunosuppressive tumor microenvironment and the dynamic nature of leukemic clonal architecture.

For patients in remission or with low disease burden, neoepitope-based vaccines or adoptive T cell therapies may offer an opportunity to eliminate MRD and prolong survival. Nonetheless, monotherapy approaches targeting neoantigens are unlikely to suffice. Combination strategies—pairing neoepitope-based interventions with checkpoint blockade, metabolic modulators, or cytokine support—will likely be necessary to overcome the barriers imposed by the AML TME. In conclusion, while neoepitope-targeted immunotherapy represents a conceptually appealing avenue for AML treatment, its translation into clinical practice is fraught with challenges. A balanced assessment must recognize both its potential and its limitations. Future studies should prioritize rigorous validation of immunogenic peptides, explore inter-patient variability, and incorporate strategies to overcome T cell exhaustion and antigenic heterogeneity. Only through such integrative approaches can the promise of personalized immunotherapy in AML be fully realized.

## 6 Conclusions

This review underscores the multifaceted complexity of the immune microenvironment in AML, highlighting its dynamic and heterogeneous nature across genetic and clinical contexts. Genetic mutations in AML not only alter the intrinsic behavior of leukemic cells but also remodel the surrounding immune milieu—affecting immune cell infiltration, polarization, and effector function. These alterations underlie diverse immune evasion mechanisms that contribute to immunosuppression, disease progression, and treatment resistance. Importantly, these immunologic changes are not uniform but vary significantly across ELN-defined genetic risk categories, with low-risk mutations often associated with more immunologically active profiles, and high-risk mutations linked to profound immune dysfunction. This suggests that effective therapeutic strategies must account for both genetic and immune stratification. While traditional chemotherapy remains the cornerstone of AML treatment, its efficacy is limited, particularly in patients harboring high-risk molecular lesions. Recent progress in targeted therapies and immunotherapies—especially those directed against neoepitopes derived from AML driver mutations—has provided new hope for achieving disease control. However, multiple barriers remain, including immune exhaustion, neoepitope heterogeneity, and the deeply immunosuppressive bone marrow microenvironment. Future research should therefore prioritize the development of integrative treatment strategies that not only target specific genetic lesions but also modulate the immune contexture. Combining neoepitope-based interventions with immune checkpoint blockade, T cell engineering, or microenvironment-modulating agents may help overcome resistance and enhance long-term therapeutic responses. Ultimately, the advancement of personalized immunotherapy—guided by both molecular and immunologic profiling—holds the greatest promise for improving outcomes in AML.

## References

[1] Reis R Müller GS Santos MM Santos AS Santos H Santos LS. Description of lymphocyte and cytokine profiles in individuals with acute myeloid leukemia associated with FLT3-ITD and NPM1 mutation status. Eur J Cancer Prev. (2024) 34(2):115–23. doi: 10.1097/CEJ.0000000000000905, PMID: 38904445

[2] Prada-Arismendy J Arroyave JC Röthlisberger S. Molecular biomarkers in acute myeloid leukemia. Blood Rev. (2017) 31:63–76. doi: 10.1016/j.blre.2016.08.005, PMID: 27639498

[3] Arber DA Orazi A Hasserjian R Thiele J Borowitz MJ Le Beau MM. The 2016 revision to the World Health Organization classification of myeloid neoplasms and acute leukemia. Blood. (2016) 127:2391–405. doi: 10.1182/blood-2016-03-643544, PMID: 27069254

[4] Mumme H Thomas BE Bhasin SS Krishnan U Dwivedi B Perumalla P. Single-cell analysis reveals altered tumor microenvironments of relapse- and remission-associated pediatric acute myeloid leukemia. Nat Commun. (2023) 14:6209. doi: 10.1038/s41467-023-41994-0, PMID: 37798266 PMC10556066

[5] Xie G Ivica NA Jia B Li Y Dong H Liang Y. CAR-T cells targeting a nucleophosmin neoepitope exhibit potent specific activity in mouse models of acute myeloid leukaemia. Nat Biomed engineering. (2021) 5:399–413. doi: 10.1038/s41551-020-00625-5, PMID: 33046866 PMC8039062

[6] Zhao Y Chen W Yu J Pei S Zhang Q Shi J. TP53 in MDS and AML: Biological and clinical advances. Cancer letters. (2024) 588:216767. doi: 10.1016/j.canlet.2024.216767, PMID: 38417666

[7] Park SH Bae MH Park CJ Cho YU Jang S Lee JH. Effect of changes in lymphocyte subsets at diagnosis in acute myeloid leukemia on prognosis: association with complete remission rates and relapse free survivals. J hematopathology. (2023) 16:73–84. doi: 10.1007/s12308-023-00536-9, PMID: 38175440

[8] Cianga VA Rusu C Pavel-Tanasa M Dascalescu A Danaila C Harnau S. Combined flow cytometry natural killer immunophenotyping and KIR/HLA-C genotyping reveal remarkable differences in acute myeloid leukemia patients, but suggest an overall impairment of the natural killer response. Front Med. (2023) 10:1148748. doi: 10.3389/fmed.2023.1148748, PMID: 36960339 PMC10028202

[9] Cianga VA Campos Catafal L Cianga P Pavel Tanasa M Cherry M Collet P. Natural killer cell subpopulations and inhibitory receptor dynamics in myelodysplastic syndromes and acute myeloid leukemia. Front Immunol. (2021) 12:665541. doi: 10.3389/fimmu.2021.665541, PMID: 33986753 PMC8112610

[10] Leifheit ME Johnson G Kuzel TM Schneider JR Barker E Yun HD. Enhancing therapeutic efficacy of FLT3 inhibitors with combination therapy for treatment of acute myeloid leukemia. Int J Mol Sci. (2024) 25(17):9448. doi: 10.3390/ijms25179448, PMID: 39273395 PMC11394928

[11] Straube J Janardhanan Y Haldar R Bywater MJ. Immune control in acute myeloid leukemia. Exp hematology. (2024) 138:104256. doi: 10.1016/j.exphem.2024.104256, PMID: 38876254

[12] Wu Z Zhang H Wu M Peng G He Y Wan N. Targeting the NKG2D/NKG2D-L axis in acute myeloid leukemia. Biomedicine pharmacotherapy = Biomedecine pharmacotherapie. (2021) 137:111299. doi: 10.1016/j.biopha.2021.111299, PMID: 33508619

[13] Chretien AS Devillier R Granjeaud S Cordier C Demerle C Salem N. High-dimensional mass cytometry analysis of NK cell alterations in AML identifies a subgroup with adverse clinical outcome. Proc Natl Acad Sci United States America. (2021) 118(22):e2020459118. doi: 10.1073/pnas.2020459118, PMID: 34050021 PMC8179170

[14] Brauneck F Fischer B Witt M Muschhammer J Oelrich J da Costa Avelar PH. TIGIT blockade repolarizes AML-associated TIGIT(+) M2 macrophages to an M1 phenotype and increases CD47-mediated phagocytosis. J immunotherapy Cancer. (2022) 10(12):e004794. doi: 10.1136/jitc-2022-004794, PMID: 36549780 PMC9791419

[15] Xu L Liu L Yao D Zeng X Zhang Y Lai J. PD-1 and TIGIT are highly co-expressed on CD8(+) T cells in AML patient bone marrow. Front Oncol. (2021) 11:686156. doi: 10.3389/fonc.2021.686156, PMID: 34490086 PMC8416522

[16] Flynn PA Long MD Kosaka Y Long N Mulkey JS Coy JL. Leukemic mutation FLT3-ITD is retained in dendritic cells and disrupts their homeostasis leading to expanded Th17 frequency. Front Immunol. (2024) 15:1297338. doi: 10.3389/fimmu.2024.1297338, PMID: 38495876 PMC10943691

[17] Jia B Wang L Claxton DF Ehmann WC Rybka WB Mineishi S. Bone marrow CD8 T cells express high frequency of PD-1 and exhibit reduced anti-leukemia response in newly diagnosed AML patients. Blood Cancer J. (2018) 8:34. doi: 10.1038/s41408-018-0069-4, PMID: 29563517 PMC5862839

[18] Le Dieu R Taussig DC Ramsay AG Mitter R Miraki-Moud F Fatah R. Peripheral blood T cells in acute myeloid leukemia (AML) patients at diagnosis have abnormal phenotype and genotype and form defective immune synapses with AML blasts. Blood. (2009) 114:3909–16. doi: 10.1182/blood-2009-02-206946, PMID: 19710498 PMC2773481

[19] Knaus HA Berglund S Hackl H Blackford AL Zeidner JF Montiel-Esparza R. Signatures of CD8+ T cell dysfunction in AML patients and their reversibility with response to chemotherapy. JCI Insight. (2018) 3(21):e120974. doi: 10.1172/jci.insight.120974, PMID: 30385732 PMC6238744

[20] Brück O Dufva O Hohtari H Blom S Turkki R Ilander M. Immune profiles in acute myeloid leukemia bone marrow associate with patient age, T-cell receptor clonality, and survival. Blood advances. (2020) 4:274–86. doi: 10.1182/bloodadvances.2019000792, PMID: 31968078 PMC6988390

[21] Hao F Sholy C Wang C Cao M Kang X. The role of T cell immunotherapy in acute myeloid leukemia. Cells. (2021) 10(12):3376. doi: 10.3390/cells10123376, PMID: 34943884 PMC8699747

[22] Luciano M Krenn PW Horejs-Hoeck J. The cytokine network in acute myeloid leukemia. Front Immunol. (2022) 13:1000996. doi: 10.3389/fimmu.2022.1000996, PMID: 36248849 PMC9554002

[23] Forsberg M Konopleva M. SOHO state of the art updates and next questions: understanding and overcoming venetoclax resistance in hematologic Malignancies. Clin lymphoma myeloma leukemia. (2024) 24:1–14. doi: 10.1016/j.clml.2023.10.006, PMID: 38007372

[24] Zhong F Yao F Jiang J Yu X Liu J Huang B. CD8 + T cell-based molecular subtypes with heterogeneous immune landscapes and clinical significance in acute myeloid leukemia. Inflammation Res. (2024) 73:329–44. doi: 10.1007/s00011-023-01839-4, PMID: 38195768

[25] You X Liu F Binder M Vedder A Lasho T Wen Z. Asxl1 loss cooperates with oncogenic Nras in mice to reprogram the immune microenvironment and drive leukemic transformation. Blood. (2022) 139:1066–79. doi: 10.1182/blood.2021012519, PMID: 34699595 PMC8854684

[26] Williams P Basu S Garcia-Manero G Hourigan CS Oetjen KA Cortes JE. The distribution of T-cell subsets and the expression of immune checkpoint receptors and ligands in patients with newly diagnosed and relapsed acute myeloid leukemia. Cancer. (2019) 125:1470–81. doi: 10.1002/cncr.31896, PMID: 30500073 PMC6467779

[27] El Dosoky W Aref S El Menshawy N Ramez A Abou Zaid T Aref M. Prognostic effect of CTLA4/LAG3 expression by T-cells subsets on acute myeloid leukemia patients. Asian Pacific J Cancer prevention: APJCP. (2024) 25:1777–85. doi: 10.31557/APJCP.2024.25.5.1777, PMID: 38809650 PMC11318815

[28] Sun K Shi ZY Wang YZ Xie DH Liu YR Jiang Q. The profile and prognostic significance of bone marrow T-cell differentiation subsets in adult AML at diagnosis. Front Immunol. (2024) 15:1418792. doi: 10.3389/fimmu.2024.1418792, PMID: 39100667 PMC11294180

[29] Shapoorian H Zalpoor H Ganjalikhani-Hakemi M. The correlation between Flt3-ITD mutation in dendritic cells with TIM-3 expression in acute myeloid leukemia. Blood Sci (Baltimore Md). (2021) 3:132–5. doi: 10.1097/BS9.0000000000000092, PMID: 35402842 PMC8975045

[30] Lasry A Nadorp B Fornerod M Nicolet D Wu H Walker CJ. An inflammatory state remodels the immune microenvironment and improves risk stratification in acute myeloid leukemia. Nat cancer. (2023) 4:27–42. doi: 10.1038/s43018-022-00480-0, PMID: 36581735 PMC9986885

[31] Rausch C Rothenberg-Thurley M Dufour A Schneider S Gittinger H Sauerland C. Validation and refinement of the 2022 European LeukemiaNet genetic risk stratification of acute myeloid leukemia. Leukemia. (2023) 37:1234–44. doi: 10.1038/s41375-023-01884-2, PMID: 37041198 PMC10244159

[32] Döhner H DiNardo CD Wei AH Löwenberg B Appelbaum F Craddock C. Genetic risk classification for adults with AML receiving less-intensive therapies: the 2024 ELN recommendations. Blood. (2024) 144(21):2169–73. doi: 10.1182/blood.2024025409, PMID: 39133932

[33] Chen H Wu M Xia H Du S Zhou G Long G. FLT3LG and IFITM3P6 consolidate T cell activity in the bone marrow microenvironment and are prognostic factors in acute myelocytic leukemia. Front Immunol. (2022) 13:980911. doi: 10.3389/fimmu.2022.980911, PMID: 36081495 PMC9445253

[34] Tecik M Adan A. Emerging DNA methylome targets in FLT3-ITD-positive acute myeloid leukemia: combination therapy with clinically approved FLT3 inhibitors. Curr Treat options Oncol. (2024) 25:719–51. doi: 10.1007/s11864-024-01202-7, PMID: 38696033 PMC11222205

[35] Comont T Nicolau-Travers ML Bertoli S Recher C Vergez F Treiner E. MAIT cells numbers and frequencies in patients with acute myeloid leukemia at diagnosis: association with cytogenetic profile and gene mutations. Cancer immunology immunotherapy: CII. (2022) 71:875–87. doi: 10.1007/s00262-021-03037-9, PMID: 34477901 PMC10991316

[36] Lau CM Nish SA Yogev N Waisman A Reiner SL Reizis B. Leukemia-associated activating mutation of Flt3 expands dendritic cells and alters T cell responses. J Exp Med. (2016) 213:415–31. doi: 10.1084/jem.20150642, PMID: 26903243 PMC4813676

[37] Hu Z Yang Y Li J Hu Z. Genetic mutations and immune microenvironment: unveiling the connection to AML prognosis. Hematol (Amsterdam Netherlands). (2024) 29:2346965. doi: 10.1080/16078454.2024.2346965, PMID: 38687637

[38] Kaito Y Hirano M Futami M Nojima M Tamura H Tojo A. CD155 and CD112 as possible therapeutic targets of FLT3 inhibitors for acute myeloid leukemia. Oncol letters. (2022) 23:51. doi: 10.3892/ol.2021.13169, PMID: 34992684 PMC8721849

[39] Chen C Liang C Wang S Chio CL Zhang Y Zeng C. Expression patterns of immune checkpoints in acute myeloid leukemia. J Hematol Oncol. (2020) 13:28. doi: 10.1186/s13045-020-00853-x, PMID: 32245463 PMC7118887

[40] Brodská B Otevřelová P Šálek C Fuchs O Gašová Z Kuželová K. High PD-L1 expression predicts for worse outcome of leukemia patients with concomitant NPM1 and FLT3 mutations. Int J Mol Sci. (2019) 20(11):2823. doi: 10.3390/ijms20112823, PMID: 31185600 PMC6600137

[41] Mendez LM Posey RR Pandolfi PP. The interplay between the genetic and immune landscapes of AML: mechanisms and implications for risk stratification and therapy. Front Oncol. (2019) 9:1162. doi: 10.3389/fonc.2019.01162, PMID: 31781488 PMC6856667

[42] Kupsa T Vanek J Zak P Jebavy L Horacek JM. Serum levels of selected cytokines and soluble adhesion molecules in acute myeloid leukemia: Soluble receptor for interleukin-2 predicts overall survival. Cytokine. (2020) 128:155005. doi: 10.1016/j.cyto.2020.155005, PMID: 32006876

[43] Zhang Q Falqués-Costa T Pilheden M Sturesson H Ovlund T Rissler V. Activating mutations remodel the chromatin accessibility landscape to drive distinct regulatory networks in KMT2A-rearranged acute leukemia. HemaSphere. (2024) 8:e70006. doi: 10.1002/hem3.70006, PMID: 39329074 PMC11426354

[44] Peng L Wei Y Shao Y Li Y Liu N Duan L. The emerging roles of CCN3 protein in immune-related diseases. Mediators inflammation. (2021) 2021:5576059. doi: 10.1155/2021/5576059, PMID: 34393649 PMC8356028

[45] Naughton M Moffat J Eleftheriadis G de la Vega Gallardo N Young A Falconer J. CCN3 is dynamically regulated by treatment and disease state in multiple sclerosis. J neuroinflammation. (2020) 17:349. doi: 10.1186/s12974-020-02025-7, PMID: 33222687 PMC7681974

[46] Guzylack-Piriou L Gausseres B Tasca C Hassel C Tabouret G Foucras G. A loss of function mutation in SOCS2 results in increased inflammatory response of macrophages to TLR ligands and Staphylococcus aureus. Front Immunol. (2024) 15:1397330. doi: 10.3389/fimmu.2024.1397330, PMID: 39185412 PMC11341364

[47] Knosp CA Schiering C Spence S Carroll HP Nel HJ Osbourn M. Regulation of Foxp3+ inducible regulatory T cell stability by SOCS2. J Immunol (Baltimore Md: 1950). (2013) 190:3235–45. doi: 10.4049/jimmunol.1201396, PMID: 23455506 PMC3607399

[48] Sarajlic M Neuper T Föhrenbach Quiroz KT Michelini S Vetter J Schaller S. IL-1β Induces SOCS2 expression in human dendritic cells. Int J Mol Sci. (2019) 20(23):5931. doi: 10.3390/ijms20235931, PMID: 31775389 PMC6928683

[49] Peng Q Huang R Wang H Xiao H Wang Y Zhai Z. Immune characteristics and prognostic implications of mucosal-associated invariant T cells in acute myeloid leukemia. Cancer immunology immunotherapy: CII. (2023) 72:4399–414. doi: 10.1007/s00262-023-03574-5, PMID: 37932426 PMC10991463

[50] Issa GC Bidikian A Venugopal S Konopleva M DiNardo CD Kadia TM. Clinical outcomes associated with NPM1 mutations in patients with relapsed or refractory AML. Blood advances. (2023) 7:933–42. doi: 10.1182/bloodadvances.2022008316, PMID: 36322818 PMC10027507

[51] Ranieri R Pianigiani G Sciabolacci S Perriello VM Marra A Cardinali V. Current status and future perspectives in targeted therapy of NPM1-mutated AML. Leukemia. (2022) 36:2351–67. doi: 10.1038/s41375-022-01666-2, PMID: 36008542 PMC9522592

[52] van der Lee DI Reijmers RM Honders MW Hagedoorn RS de Jong RC Kester MG. Mutated nucleophosmin 1 as immunotherapy target in acute myeloid leukemia. J Clin Invest. (2019) 129:774–85. doi: 10.1172/JCI97482, PMID: 30640174 PMC6355238

[53] Peng M Ren J Jing Y Jiang X Xiao Q Huang J. Tumour-derived small extracellular vesicles suppress CD8+ T cell immune function by inhibiting SLC6A8-mediated creatine import in NPM1-mutated acute myeloid leukaemia. J extracellular vesicles. (2021) 10:e12168. doi: 10.1002/jev2.12168, PMID: 34807526 PMC8607980

[54] Forghieri F Riva G Lagreca I Barozzi P Bettelli F Paolini A. Neoantigen-Specific T-cell immune responses: the paradigm of NPM1-mutated acute myeloid leukemia. Int J Mol Sci. (2021) 22(17):9159. doi: 10.3390/ijms22179159, PMID: 34502069 PMC8431540

[55] Antohe I Dǎscǎlescu A Dǎnǎilǎ C Titieanu A Zlei M Ivanov I. B7-positive and B7-negative acute myeloid leukemias display distinct T cell maturation profiles, immune checkpoint receptor expression, and european leukemia net risk profiles. Front Oncol. (2020) 10:264. doi: 10.3389/fonc.2020.00264, PMID: 32231996 PMC7082324

[56] Greiner J Goetz M Schuler PJ Bulach C Hofmann S Schrezenmeier H. Enhanced stimulation of antigen-specific immune responses against nucleophosmin 1 mutated acute myeloid leukaemia by an anti-programmed death 1 antibody. Br J haematology. (2022) 198:866–74. doi: 10.1111/bjh.18326, PMID: 35799423

[57] Dong Y Han Y Huang Y Jiang S Huang Z Chen R. PD-L1 is expressed and promotes the expansion of regulatory T cells in acute myeloid leukemia. Front Immunol. (2020) 11:1710. doi: 10.3389/fimmu.2020.01710, PMID: 32849603 PMC7412746

[58] Kuželová K Brodská B Marková J Petráčková M Schetelig J Ransdorfová Š. NPM1 and DNMT3A mutations are associated with distinct blast immunophenotype in acute myeloid leukemia. Oncoimmunology. (2022) 11:2073050. doi: 10.1080/2162402X.2022.2073050, PMID: 35558161 PMC9090295

[59] Kikushige Y Miyamoto T. TIM-3 as a novel therapeutic target for eradicating acute myelogenous leukemia stem cells. Int J hematology. (2013) 98:627–33. doi: 10.1007/s12185-013-1433-6, PMID: 24046178

[60] Zhang X Zhang X Liu P Liu K Li W Chen Q. Prognostic implications and functional enrichment analysis of LTB4R in patients with acute myeloid leukemia. Nan fang yi ke da xue xue bao = J South Med University. (2022) 42:309–20. doi: 10.12122/j.issn.1673-4254.2022.03.01, PMID: 35426793 PMC9010981

[61] Pitts HA Cheng CK Cheung JS Sun MK Yung YL Chan HY. SPINK2 protein expression is an independent adverse prognostic marker in AML and is potentially implicated in the regulation of ferroptosis and immune response. Int J Mol Sci. (2023) 24(11):9696. doi: 10.3390/ijms24119696, PMID: 37298647 PMC10253579

[62] Que Y Li H Lin L Zhu X Xiao M Wang Y. Study on the immune escape mechanism of acute myeloid leukemia with DNMT3A mutation. Front Immunol. (2021) 12:653030. doi: 10.3389/fimmu.2021.653030, PMID: 34093541 PMC8173207

[63] Jafari PA Bagheri R Lavasani S Goudarzi S. DNMT3A-R882: a mutation with many paradoxes. Ann Hematol. (2024) 103(12):4981–8. doi: 10.1007/s00277-024-05874-x, PMID: 38969930

[64] Guryanova OA Shank K Spitzer B Luciani L Koche RP Garrett-Bakelman FE. DNMT3A mutations promote anthracycline resistance in acute myeloid leukemia via impaired nucleosome remodeling. Nat Med. (2016) 22:1488–95. doi: 10.1038/nm.4210, PMID: 27841873 PMC5359771

[65] Chu X Zhong L Dan W Wang X Zhang Z Liu Z. DNMT3A R882H mutation drives daunorubicin resistance in acute myeloid leukemia via regulating NRF2/NQO1 pathway. Cell communication signaling: CCS. (2022) 20:168. doi: 10.1186/s12964-022-00978-1, PMID: 36303144 PMC9615155

[66] Elsayed GM Abd Elgawad AF Shafik NF Elshimy RA Abd Elhakeem HK Attea SA. Study of DNA methyl transferase 3A mutation in acute myeloid leukemic patients. Egyptian J Med Human Genet. (2018) 19:315–9. doi: 10.1016/j.ejmhg.2018.05.005

[67] Wei X Huang S Gu Z Liu J Li M Jin X. Clonal hematopoiesis-associated gene mutations affect acute graft-versus-host disease after hematopoietic stem cell transplantation in AML patients. Ann transplantation. (2024) 29:e943688. doi: 10.12659/AOT.943688, PMID: 38952007 PMC11299484

[68] Ehx G Larouche JD Durette C Laverdure JP Hesnard L Vincent K. Atypical acute myeloid leukemia-specific transcripts generate shared and immunogenic MHC class-I-associated epitopes. Immunity. (2021) 54:737–52.e10. doi: 10.1016/j.immuni.2021.03.001, PMID: 33740418

[69] Gamlen HA Romer-Seibert JS Lawler ME Versace AM Goetz ML Feng Y. miR-196b-TLR7/8 signaling axis regulates innate immune signaling and myeloid maturation in DNMT3A-mutant AML. Clin Cancer Res. (2022) 28:4574–86. doi: 10.1158/1078-0432.CCR-22-1598, PMID: 35943291 PMC9588567

[70] Xu Q Guo T. Somatic mutation-associated risk index based on lncRNA expression for predicting prognosis in acute myeloid leukemia. Hematol (Amsterdam Netherlands). (2022) 27:659–71. doi: 10.1080/16078454.2022.2056677, PMID: 35666642

[71] Zeidan AM Bewersdorf JP Hasle V Shallis RM Thompson E de Menezes DL. Integrated genetic, epigenetic, and immune landscape of TP53 mutant AML and higher risk MDS treated with azacitidine. Ther Adv hematology. (2024) 15:20406207241257904. doi: 10.1177/20406207241257904, PMID: 38883163 PMC11180421

[72] Bai L Hao X Keith J Feng Y. DNA methylation in regulatory T cell differentiation and function: challenges and opportunities. Biomolecules. (2022) 12(9):1282. doi: 10.3390/biom12091282, PMID: 36139121 PMC9496199

[73] Zhu G Cai J Fu W Sun Y Wang T Zhong H. Elucidating the immune landscape and potential prognostic model in acute myeloid leukemia with TP53 mutation. Hematol (Amsterdam Netherlands). (2024) 29:2400620. doi: 10.1080/16078454.2024.2400620, PMID: 39327848

[74] Vadakekolathu J Lai C Reeder S Church SE Hood T Lourdusamy A. TP53 abnormalities correlate with immune infiltration and associate with response to flotetuzumab immunotherapy in AML. Blood advances. (2020) 4:5011–24. doi: 10.1182/bloodadvances.2020002512, PMID: 33057635 PMC7594389

[75] Urabe A Chi S Minami Y. The immuno-oncology and genomic aspects of DNA-hypomethylating therapeutics in acute myeloid leukemia. Int J Mol Sci. (2023) 24(4):3727. doi: 10.3390/ijms24043727, PMID: 36835136 PMC9961620

[76] Chomczyk M Gazzola L Dash S Firmanty P George BS Mohanty V. Impact of p53-associated acute myeloid leukemia hallmarks on metabolism and the immune environment. Front Pharmacol. (2024) 15:1409210. doi: 10.3389/fphar.2024.1409210, PMID: 39161899 PMC11330794

[77] Daver NG Maiti A Kadia TM Vyas P Majeti R Wei AH. TP53-mutated myelodysplastic syndrome and acute myeloid leukemia: biology, current therapy, and future directions. Cancer discovery. (2022) 12:2516–29. doi: 10.1158/2159-8290.CD-22-0332, PMID: 36218325 PMC9627130

[78] Sallman DA McLemore AF Aldrich AL Komrokji RS McGraw KL Dhawan A. TP53 mutations in myelodysplastic syndromes and secondary AML confer an immunosuppressive phenotype. Blood. (2020) 136:2812–23. doi: 10.1182/blood.2020006158, PMID: 32730593 PMC7731792

[79] Abolhalaj M Sincic V Lilljebjörn H Sandén C Aab A Hägerbrand K. Transcriptional profiling demonstrates altered characteristics of CD8(+) cytotoxic T-cells and regulatory T-cells in TP53-mutated acute myeloid leukemia. Cancer Med. (2022) 11:3023–32. doi: 10.1002/cam4.4661, PMID: 35297213 PMC9359873

[80] Wen XM Xu ZJ Jin Y Xia PH Ma JC Qian W. Association analyses of TP53 mutation with prognosis, tumor mutational burden, and immunological features in acute myeloid leukemia. Front Immunol. (2021) 12:717527. doi: 10.3389/fimmu.2021.717527, PMID: 34745095 PMC8566372

[81] Shallis RM Bewersdorf JP Stahl MF Halene S Zeidan AM. Are we moving the needle for patients with TP53-mutated acute myeloid leukemia? Cancers. (2022) 14(10):2434. doi: 10.3390/cancers14102434, PMID: 35626039 PMC9140008

[82] Brummer T Zeiser R. The role of the MDM2/p53 axis in antitumor immune responses. Blood. (2024) 143:2701–9. doi: 10.1182/blood.2023020731, PMID: 37467495 PMC11251213

[83] Palm-Apergi C. PLK4, a potential target against AML. Blood. (2023) 142:1941–2. doi: 10.1182/blood.2023021950, PMID: 38060272

[84] Sunthankar KI Jenkins MT Cote CH Patel SB Welner RS Ferrell PB. Isocitrate dehydrogenase mutations are associated with altered IL-1β responses in acute myeloid leukemia. Leukemia. (2022) 36:923–34. doi: 10.1038/s41375-021-01487-9, PMID: 34857894 PMC9066619

[85] Galluzzi L Kroemer G. Potent immunosuppressive effects of the oncometabolite R-2-hydroxyglutarate. Oncoimmunology. (2018) 7:e1528815. doi: 10.1080/2162402X.2018.1528815, PMID: 30524910 PMC6279319

[86] Hammon K Renner K Althammer M Voll F Babl N Decking SM. D-2-hydroxyglutarate supports a tolerogenic phenotype with lowered major histocompatibility class II expression in non-malignant dendritic cells and acute myeloid leukemia cells. Haematologica. (2024) 109:2500–14. doi: 10.3324/haematol.2023.283597, PMID: 38235501 PMC11290548

[87] Testa U Castelli G Pelosi E. TP53-mutated myelodysplasia and acute myeloid leukemia. Mediterr J Hematol Infect diseases. (2023) 15:e2023038. doi: 10.4084/MJHID.2023.038, PMID: 37435040 PMC10332352

[88] Mustafa Ali MK Williams MT Corley EM AlKaabba F Niyongere S. Impact of KRAS and NRAS mutations on outcomes in acute myeloid leukemia. Leukemia lymphoma. (2023) 64:962–71. doi: 10.1080/10428194.2023.2190432, PMID: 37042657

[89] Austin RJ Straube J Halder R Janardhanan Y Bruedigam C Witkowski M. Oncogenic drivers dictate immune control of acute myeloid leukemia. Nat Commun. (2023) 14:2155. doi: 10.1038/s41467-023-37592-9, PMID: 37059710 PMC10104832

[90] Hinshaw DC Shevde LA. The tumor microenvironment innately modulates cancer progression. Cancer Res. (2019) 79:4557–66. doi: 10.1158/0008-5472.CAN-18-3962, PMID: 31350295 PMC6744958

[91] Lyu A Nam SH Humphrey RS Horton TM Ehrlich LIR. Cells and signals of the leukemic microenvironment that support progression of T-cell acute lymphoblastic leukemia (T-ALL). Exp Mol Med. (2024) 56(11):2337–47. doi: 10.1038/s12276-024-01335-7, PMID: 39482533 PMC11612169

[92] Menter T Tzankov A. Tumor microenvironment in acute myeloid leukemia: adjusting niches. Front Immunol. (2022) 13:811144. doi: 10.3389/fimmu.2022.811144, PMID: 35273598 PMC8901718

[93] Chen T Zhang Y Zhang D Zhou H. Immune-based subgroups uncover diverse tumor immunogenicity and implications for prognosis and precision therapy in acute myeloid leukemia. Front Immunol. (2024) 15:1451486. doi: 10.3389/fimmu.2024.1451486, PMID: 39582863 PMC11581856

[94] Nelde A Schuster H Heitmann JS Bauer J Maringer Y Zwick M. Immune surveillance of acute myeloid leukemia is mediated by HLA-presented antigens on leukemia progenitor cells. Blood Cancer discovery. (2023) 4:468–89. doi: 10.1158/2643-3230.BCD-23-0020, PMID: 37847741 PMC10618727

[95] Forghieri F Riva G Lagreca I Barozzi P Vallerini D Morselli M. Characterization and dynamics of specific T cells against nucleophosmin-1 (NPM1)-mutated peptides in patients with NPM1-mutated acute myeloid leukemia. Oncotarget. (2019) 10:869–82. doi: 10.18632/oncotarget.26617, PMID: 30783516 PMC6368236

[96] Wei X Li Y Zhang G Wang N Mi M Xin Y. IL-37 was involved in progress of acute myeloid leukemia through regulating IL-6 expression. Cancer Manage Res. (2021) 13:3393–402. doi: 10.2147/CMAR.S303017, PMID: 33907463 PMC8064683

[97] Radpour R Riether C Simillion C Höpner S Bruggmann R Ochsenbein AF. CD8(+) T cells expand stem and progenitor cells in favorable but not adverse risk acute myeloid leukemia. Leukemia. (2019) 33:2379–92. doi: 10.1038/s41375-019-0441-9, PMID: 30877275

[98] Kang TG Lan X Mi T Chen H Alli S Lim SE. Epigenetic regulators of clonal hematopoiesis control CD8 T cell stemness during immunotherapy. Sci (New York NY). (2024) 386:eadl4492. doi: 10.1126/science.adl4492, PMID: 39388542 PMC11697317

[99] Lee JB Khan DH Hurren R Xu M Na Y Kang H. Venetoclax enhances T cell-mediated antileukemic activity by increasing ROS production. Blood. (2021) 138:234–45. doi: 10.1182/blood.2020009081, PMID: 34292323 PMC8310428

[100] Saadh MJ Torabi Fard N Hussein A Mirzazadeh A Siavashi M SeyedMoharami F. Mesenchymal stem cells in the bone marrow microenvironment: a double-edged sword for AML. J Cancer Res Clin Oncol. (2025) 151:193. doi: 10.1007/s00432-025-06244-4, PMID: 40542231 PMC12181099

[101] Jin P Shen J Zhao M Yu J Jin W Jiang G. Driver mutation landscape of acute myeloid leukemia provides insights for neoantigen-based immunotherapy. Cancer letters. (2024) 611:217427. doi: 10.1016/j.canlet.2024.217427, PMID: 39725148

[102] Sahib N Mohamed JS Rashid M Jayalakshmi LYC Lin YC Chee YL. A combinatorial functional precision medicine platform for rapid therapeutic response prediction in AML. Cancer Med. (2024) 13:e70401. doi: 10.1002/cam4.70401, PMID: 39560206 PMC11574777

[103] Tettamanti S Pievani A Biondi A Dotti G Serafini M. Catch me if you can: how AML and its niche escape immunotherapy. Leukemia. (2022) 36:13–22. doi: 10.1038/s41375-021-01350-x, PMID: 34302116 PMC8727297

[104] Goulart H Wei AH Kadia TM. Maintenance therapy in AML: what is the future potential? Am J Hematol. (2025) 100 Suppl 2:38–49. doi: 10.1002/ajh.27583, PMID: 39960005

[105] Kantarjian HM DiNardo CD Kadia TM Daver NG Altman JK Stein EM. Acute myeloid leukemia management and research in 2025. CA Cancer J Clin. (2025) 75:46–67. doi: 10.3322/caac.21873, PMID: 39656142 PMC11745214

[106] Saadh MJ KA W Ashurova D Sanghvi G Ballal S Sharma R. FLT3-mutated AML: immune evasion through exosome-mediated mechanisms and innovative combination therapies targeting immune escape. Expert Rev Anticancer Ther. (2025) 25:143–50. doi: 10.1080/14737140.2025.2461632, PMID: 39885639

[107] Bruzzese A Martino EA Labanca C Mendicino F Lucia E Olivito V. Advances and challenges in quizartinib-based FLT3 inhibition for acute myeloid leukemia: mechanisms of resistance and prospective combination therapies. Eur J Haematol. (2025) 114(4):584–95. doi: 10.1111/ejh.14383, PMID: 39763167 PMC11880963

[108] Yamaguchi H. Advances in pathogenesis research and challenges in treatment development for acute myeloid leukemia. Int J hematology. (2024) 120:414–6. doi: 10.1007/s12185-024-03837-6, PMID: 39225969

[109] Zhang Y Zhang Z. The history and advances in cancer immunotherapy: understanding the characteristics of tumor-infiltrating immune cells and their therapeutic implications. Cell Mol Immunol. (2020) 17:807–21. doi: 10.1038/s41423-020-0488-6, PMID: 32612154 PMC7395159

[110] Greiner J Ono Y Hofmann S Schmitt A Mehring E Götz M. Mutated regions of nucleophosmin 1 elicit both CD4(+) and CD8(+) T-cell responses in patients with acute myeloid leukemia. Blood. (2012) 120:1282–9. doi: 10.1182/blood-2011-11-394395, PMID: 22592607

[111] Struckman NE de Jong RCM Honders MW Smith SI van der Lee DI Koutsoumpli G. Hotspot DNA methyltransferase 3A (DNMT3A) and isocitrate dehydrogenase 1 and 2 (IDH1/2) mutations in acute myeloid leukemia and their relevance as targets for immunotherapy. Biomedicines. (2024) 12(5):1086. doi: 10.3390/biomedicines12051086, PMID: 38791049 PMC11118067

[112] Greiner J Schneider V Schmitt M Gotz M Dohner K Wiesneth M. Immune responses against the mutated region of cytoplasmatic NPM1 might contribute to the favorable clinical outcome of AML patients with NPM1 mutations (NPM1mut). Blood. (2013) 122:1087–8. doi: 10.1182/blood-2013-04-496844, PMID: 23929838

[113] Greiner J Mohamed E Fletcher DM Schuler PJ Schrezenmeier H Gotz M. Immunotherapeutic potential of mutated NPM1 for the treatment of acute myeloid leukemia. Cancers. (2024) 16(20):3443. doi: 10.3390/cancers16203443, PMID: 39456538 PMC11505958

[114] Graf C Heidel F Tenzer S Radsak MP Solem FK Britten CM. A neoepitope generated by an FLT3 internal tandem duplication (FLT3-ITD) is recognized by leukemia-reactive autologous CD8+ T cells. Blood. (2007) 109:2985–8. doi: 10.1182/blood-2006-07-032839, PMID: 17119119

[115] Giannakopoulou E Lehander M Virding Culleton S Yang W Li Y Karpanen T. A T cell receptor targeting a recurrent driver mutation in FLT3 mediates elimination of primary human acute myeloid leukemia *in vivo* . Nat Cancer. (2023) 4:1474–90. doi: 10.1038/s43018-023-00642-8, PMID: 37783807 PMC10597840

[116] Rombola G Crocchiolo R Falco M Iozzi S Marseglia G Amoriello R. Selective HLA haplotype loss in npm1-positive acute myeloid leukaemia: A model of immunological escape. Hla. (2025) 105:e70058. doi: 10.1111/tan.70058, PMID: 39933756

[117] Jung S Nelde A Maringer Y Denk M Zieschang L Kammer C. AML-VAC-XS15-01: protocol of a first-in-human clinical trial to evaluate the safety, tolerability and preliminary efficacy of a multi-peptide vaccine based on leukemia stem cell antigens in acute myeloid leukemia patients. Front Oncol. (2024) 14:1458449. doi: 10.3389/fonc.2024.1458449, PMID: 39469638 PMC11513396

[118] Vadakekolathu J Minden MD Hood T Church SE Reeder S Altmann H. Immune landscapes predict chemotherapy resistance and immunotherapy response in acute myeloid leukemia. Sci Trans Med. (2020) 12(546):eaaz0463. doi: 10.1126/scitranslmed.aaz0463, PMID: 32493790 PMC7427158

[119] Moore CG Stein A Fathi AT Pullarkat V. Treatment of relapsed/refractory AML-novel treatment options including immunotherapy. Am J hematology. (2025) 100 Suppl 2:23–37. doi: 10.1002/ajh.27584, PMID: 39960017

