# Supplementary material for: The interaction between common genetic mutations in AML and the immune landscape: mechanisms and implications for immune response
Source: Front Immunol. 2025 Aug 11;16:1635111. doi: 10.3389/fimmu.2025.1635111 (PMC12375677; doi:10.3389/fimmu.2025.1635111)
Supplement: Supplementary file 1 [file DataSheet1.pdf]

Supplementary Material

Supplementary Table 1. Common Genetic Mutations and Molecular Mechanisms in AML

Gene	Mutation Classification	Mutation Hotspot	Mutation Frequency in AML	Molecular Mechanism	Impact	Prognostic Risk Stratification(1, 2)
FLT3-ITD	Insertion Mutation(3)	Juxtamembrane(3)	~20–30%(4-6)	The FLT3-ITD mutation in exons 14 and 15 of the FLT3 gene(7), located in the juxtamembrane region of the protein, causes constitutive activation of tyrosine kinase, triggering survival pathways such as PI3K/AKT, Ras/ERK, and JAK/STAT(3, 8).	FLT3-ITD disrupts the inhibitory function of the JM domain, thus promoting FLT3 activation in the absence of FLT3 ligand(3).	Intermediate Risk

FLT3-TKD	Point Mutation /Missense Mutation (3, 7)	D835 and I836(3)	~5-10%(9, 10)	D835 and I836 mutations or I836 codon deletion lead to ligand-independent FLT3 dimerization, autophosphorylation, and constitutive activation of downstream signaling pathways(10).	FLT3-TKD mutations enhance unregulated FLT3 activation through stabilization of the active, DFG-in conformation(3).	Intermediate Risk
NPM1	Insertion Mutation /Frameshift Mutation(11)	Exon 12(12)	~20-35%(11)	The insertion of 4 base pairs at the C-terminus disrupts the nucleolar localization signal(11).	The NPM1c protein is abnormally localized to the cytoplasm(11).	Low Risk
DNMT3A	Point Mutation /Missense Mutation (13)	R882H(14)	~13%(15)	The arginine at position 882 is replaced by histidine, leading to abnormal tetramer formation and inhibition of the activity of wild-type DNMT3A(14).	Reduces methylation rate and causes abnormal DNA methylation(16).	Intermediate Risk
TP53	Point Mutation /Missense Mutation (17)	DNA-binding domain(17)	–	Mutations in the DNA-binding domain of TP53 impair its ability to transactivate multiple target genes, resulting in functional inactivation of p53 and loss of its DNA-binding(17).	Compromising p53's normal function as a tumor suppressor gene(18).	High Risk

IDH1	Point Mutation /Missense Mutation (19)	R132(20)	~3-10%(20, 21)	In the cytoplasm, mutant IDH1 converts α-KG into the oncometabolite (R)-2-HG, which competitively inhibits α-KG-dependent epigenetic regulators, resulting in genome-wide DNA and histone hypermethylation(19).	Proliferation and differentiation of immature hematopoietic cells are disrupted(19).	Low Risk
IDH2	Point Mutation /Missense Mutation (19)	R140 and R172(23)	~1-4%(21)	In the mitochondria, mutant IDH2 converts α-KG into large amounts of the oncometabolite (R)-2-HG, which competitively inhibits α-KG-dependent epigenetic regulators, leading to genome-wide DNA and histone hypermethylation(19).	Proliferation and differentiation of immature hematopoietic cells are disrupted(19).	Low Risk or High Risk
NRAS	Point Mutation(22)	G12,G13,and Q61(24, 25)	~1-4%(26)	Ras proteins remain constitutively active in their GTP-bound state, losing the ability to undergo normal GTP/GDP cycling(26).	Activates pro-proliferative and anti-apoptotic signaling pathways(26).	Intermediate Risk

frontiers

References

1. Rausch C, Rothenberg-Thurley M, Dufour A, Schneider S, Gittinger H, Sauerland C, et al. Validation and refinement of the 2022 European LeukemiaNet genetic risk stratification of acute myeloid leukemia. Leukemia. 2023;37(6):1234-44.

2. Döhner H, DiNardo CD, Wei AH, Löwenberg B, Appelbaum F, Craddock C, et al. Genetic risk classification for adults with AML receiving less-intensive therapies: the 2024 ELN recommendations. Blood. 2024.

3. Wang X, DeFilippis RA, Weldemichael T, Gunaganti N, Tran P, Leung Y-K, et al. An imidazo [1, 2-a] pyridine-pyridine derivative potently inhibits FLT3-ITD and FLT3-ITD secondary mutants, including gilteritinib-resistant FLT3-ITD/F691L. 2024;264:115977.

4. Boudry A, Darmon S, Duployez N, Figeac M, Geffroy S, Bucci M, et al. Frugal alignment-free identification of FLT3-internal tandem duplications with FiLT3r. BMC bioinformatics. 2022;23(1):448.

5. Mizuki M, Fenski R, Halfter H, Matsumura I, Schmidt R, Müller C, et al. Flt3 mutations from patients with acute myeloid leukemia induce transformation of 32D cells mediated by the Ras and STAT5 pathways. Blood. 2000;96(12):3907-14.

6. Arwanih EY, Rinaldi I, Wanandi SI, Louisa M. Identification of a novel mutation of the FLT3 gene located on the juxtamembrane domain from acute myeloid leukemia patients. Molecular biology reports. 2024;51(1):867.

7. Perrone S, Ottone T, Zhdanovskaya N, Molica M. How acute myeloid leukemia (AML) escapes from FMS-related tyrosine kinase 3 (FLT3) inhibitors? Still an overrated complication? Cancer drug resistance (Alhambra, Calif). 2023;6(2):223-38.

8. Chen Y, Zou Z, Găman MA, Xu L, Li J. NADPH oxidase mediated oxidative stress signaling in FLT3-ITD acute myeloid leukemia. Cell death discovery. 2023;9(1):208.

9. Murphy KM, Levis M, Hafez MJ, Geiger T, Cooper LC, Smith BD, et al. Detection of FLT3 internal tandem duplication and D835 mutations by a multiplex polymerase chain reaction and capillary electrophoresis assay. The Journal of molecular diagnostics : JMD. 2003;5(2):96-102.

10. Akiyama H, Umezawa Y, Ishida S, Okada K, Nogami A, Miura O. Inhibition of USP9X induces apoptosis in FLT3-ITD-positive AML cells cooperatively by inhibiting the mutant kinase through aggresomal translocation and inducing oxidative stress. Cancer letters. 2019;453:84-94.

11. Brown P, McIntyre E, Rau R, Meshinchi S, Lacayo N, Dahl G, et al. The incidence and clinical significance of nucleophosmin mutations in childhood AML. Blood. 2007;110(3):979-85.

12. Yao Y, Lin X, Wang C, Gu Y, Jin J, Zhu Y, et al. Identification of a novel NPM1 mutation in acute myeloid leukemia. Experimental hematology & oncology. 2023;12(1):87.

13. Tabatabaei T, Rezvany MR, Ghasemi B, Vafaei F, Zadeh MK, Zaker F, et al. Effect of DNMT3A R882H Hot Spot Mutations on DDX43 Promoter Methylation in Acute Myeloid Leukemia. 2024;2024(1):9625043.

14. Zhou J, Wu T, Liu WJZyxyCxzzZYYZCJoMG. Research progress on DNMT3A gene expression in Acute myeloid leukemia. 2024;41(8):1010-5.

15. Ley TJ, Ding L, Walter MJ, McLellan MD, Lamprecht T, Larson DE, et al. DNMT3A mutations in acute myeloid leukemia. 2010;363(25):2424-33.

16. Holz-Schietinger C, Matje DM, Reich NOJJoBC. Mutations in DNA methyltransferase (DNMT3A) observed in acute myeloid leukemia patients disrupt processive methylation. 2012;287(37):30941-51.

17. George B, Kantarjian H, Baran N, Krocker JD, Rios AJIjoms. TP53 in acute myeloid leukemia: molecular aspects and patterns of mutation. 2021;22(19):10782.

18. Tashakori M, Kadia T, Loghavi S, Daver N, Kanagal-Shamanna R, Pierce S, et al. TP53 copy number and protein expression inform mutation status across risk categories in acute myeloid leukemia. 2022;140(1):58-72.

19. Medeiros B, Fathi A, DiNardo C, Pollyea D, Chan S, Swords RJL. Isocitrate dehydrogenase mutations in myeloid malignancies. 2017;31(2):272-81.

20. Ali A, Gale RE, Shakoori ARJJoCB. Detection of FLT3/TKD and IDH1 mutations in Pakistani acute myeloid leukemia patients by denaturing HPLC. 2017;118(5):1174-81.

21. Zarnegar-Lumley S, Alonzo TA, Gerbing RB, Othus M, Sun Z, Ries RE, et al. Characteristics and prognostic impact of IDH mutations in AML: a COG, SWOG, and ECOG analysis. 2023;7(19):5941-53.

22. Bos JLJCr. Ras oncogenes in human cancer: a review. 1989;49(17):4682-9.

23. Qin Y, Shen K, Liu T, Ma H. Prognostic value of IDH2R140 and IDH2R172 mutations in patients with acute myeloid leukemia: a systematic review and meta-analysis. BMC cancer. 2023;23(1):527.

24. Ren J-G, Xing B, Lv K, O'Keefe RA, Wu M, Wang R, et al. RAB27B controls palmitoylation-dependent NRAS trafficking and signaling in myeloid leukemia. 2023;133(12).

25. Carratt SA, Braun TP, Coblentz C, Schonrock Z, Callahan R, Curtiss BM, et al. Mutant SETBP1 enhances NRAS-driven MAPK pathway activation to promote aggressive leukemia. 2021;35(12):3594-9.

26. Jeong JH, Park SH, Park MJ, Kim MJ, Kim KH, Park PW, et al. N-ras mutation detection by pyrosequencing in adult patients with acute myeloid leukemia at a single institution. 2013;33(3):159.
